# Upregulation of mRNA Expression of ADGRD1/GPR133 and ADGRG7/GPR128 in SARS-CoV-2-Infected Lung Adenocarcinoma Calu-3 Cells

**DOI:** 10.3390/cells13100791

**Published:** 2024-05-07

**Authors:** Sandra Žáčková, Marcela Pávová, Jana Trylčová, Jitka Chalupová, Anastasiia Priss, Ondřej Lukšan, Jan Weber

**Affiliations:** 1 Institute of Organic Chemistry and Biochemistry of the Czech Academy of Sciences, 166 10 Prague, Czech Republic; sandra.zackova@uochb.cas.cz (S.Ž.); marcela.pavova@uochb.cas.cz (M.P.); jana.trylcova@uochb.cas.cz (J.T.); jitka.chalupova@uochb.cas.cz (J.C.); anastasiia.priss@uochb.cas.cz (A.P.); ondrej.luksan@uochb.cas.cz (O.L.); 2 Department of Genetics and Microbiology, Charles University, Faculty of Sciences, 128 44 Prague, Czech Republic

**Keywords:** adhesion GPCR, mRNA expression, ADGRD1, GPR133, ADGRG7, GPR128, SARS-CoV-2, Calu-3, Caco-2

## Abstract

Adhesion G protein-coupled receptors (aGPCRs) play an important role in neurodevelopment, immune defence and cancer; however, their role throughout viral infections is mostly unexplored. We have been searching for specific aGPCRs involved in SARS-CoV-2 infection of mammalian cells. In the present study, we infected human epithelial cell lines derived from lung adenocarcinoma (Calu-3) and colorectal carcinoma (Caco-2) with SARS-CoV-2 in order to analyse changes in the level of mRNA encoding individual aGPCRs at 6 and 12 h post infection. Based on significantly altered mRNA levels, we identified four aGPCR candidates—ADGRB3/BAI3, ADGRD1/GPR133, ADGRG7/GPR128 and ADGRV1/GPR98. Of these receptors, ADGRD1/GPR133 and ADGRG7/GPR128 showed the largest increase in mRNA levels in SARS-CoV-2-infected Calu-3 cells, whereas no increase was observed with heat-inactivated SARS-CoV-2 and virus-cleared conditioned media. Next, using specific siRNA, we downregulated the aGPCR candidates and analysed SARS-CoV-2 entry, replication and infectivity in both cell lines. We observed a significant decrease in the amount of SARS-CoV-2 newly released into the culture media by cells with downregulated ADGRD1/GPR133 and ADGRG7/GPR128. In addition, using a plaque assay, we observed a reduction in SARS-CoV-2 infectivity in Calu-3 cells. In summary, our data suggest that selected aGPCRs might play a role during SARS-CoV-2 infection of mammalian cells.

## 1. Introduction

Adhesion G protein-coupled receptors (aGPCRs), the second-largest class of GPCRs, are important for organogenesis, angiogenesis, neurodevelopment, immune defence and cancer progression [1]. Currently, we recognise 33 members of the aGPCR class in humans. Although the majority are orphan receptors with unknown function, recent reports have implicated three aGPCRs in human diseases, such as Usher syndrome type II, the most common combined deafblindness [2], bilateral frontoparietal polymicrogyria, a congenital brain malformation [3], and vibratory urticaria, a skin allergy [4]. Recent advances have also linked aGPCR with numerous other pathophysiological processes, such as infertility, embryonic lethality and psychiatric disorders, as well as several tumours, including glioblastoma, leukaemia and lymphoma (reviewed in [5,6,7,8,9]).

Despite recent progress in the study of the structure, signalling and pathophysiology of aGPCRs, their possible role in viral infections is mostly unexplored. It is well established that many viruses utilise GPCR-mediated pathways to enter human cells and/or to establish infections in them. Viruses can either directly exploit GPCRs for entry, such as the chemokine CCR5 or CXCR4 coreceptors necessary for HIV-1 entry [10,11] and the serotonin receptor 5-hydroxytryptamine receptor for JC polyomavirus entry [12], or use other cell adhesion molecules such as immunoglobulin superfamily receptors (e.g., the reovirus, adenovirus, coxsackievirus and HIV-1) and integrins (e.g., the rotavirus, West Nile virus and herpes simplex virus) [11,12]. In addition, 5-hydroxytryptamine receptor antagonists and other GPCR antagonists have been found to block the entry of the Ebola and Marburg virus [13]. Moreover, beta- and gammaherpesviruses, such as the human cytomegalovirus, human herpesvirus 6 and 7, Kaposi’s sarcoma-associated herpesvirus and the Epstein–Barr virus, encode their own viral homologues of GPCRs [14,15,16,17,18]. These viral GPCRs activate a broad range of signalling pathways, causing immune evasion, cell migration stimulation and constitutive activation, leading to tumourigenesis [19,20,21,22,23,24,25]. There is currently a lack of knowledge on the role of aGPCRs in the pathogenesis of viral infections. In one study, a yeast two-hybrid screen identified human adhesion G protein-coupled receptor 125 (GPR125) interacting with the small hydrophobic protein of the mumps virus [26]. As another example, primary infection with the human cytomegalovirus induced a strong increase in G protein-coupled receptor 56 (GPR56) expression in virus-specific CD8+ T cells [27]. In addition, higher GPR56 expression in CD4+ T effector memory cells was observed in the adipose tissue of patients infected with HIV-1 [28]. G protein-coupled receptor 133 (GPR133) has so far not been implicated in viral infections, but it has been shown to play a role in the tumourigenesis of glioblastoma [29,30,31], while the dissociation of the N- and C-terminus of GPR133 leads to increased levels of cytosolic cyclic AMP [32,33,34,35]. Similarly to GPR133, G protein-coupled receptor 128 (GRP128) has also mostly been found to be connected with cancer [36,37,38].

The emergence of a third coronavirus that causes severe acute respiratory syndrome in less than two decades has exposed emerging coronaviruses as a new public health concern [39,40]. Since the first detection of SARS-CoV-2 in Wuhan in late 2019, the virus has spread in almost every country and has so far caused almost 7 million deaths from coronavirus disease 2019, commonly referred to as COVID-19 (WHO statistics). It has galvanised the research community to explore every aspect of the biology of SARS-CoV-2 in the hope of devising countermeasures against the ongoing SARS-CoV-2 pandemic and any future coronavirus epidemic. Coronaviruses, including SARS-CoV-2, can enter host cells by direct fusion with the plasma membrane or by endocytosis, depending on the membrane environment and the type of cell [41,42,43,44]. The receptor binding domain in the SARS-CoV-2 spike glycoprotein binds to the angiotensin-converting enzyme 2 (ACE-2) receptor, which is the SARS-CoV-2 entry receptor [45,46]. In the presence of membrane-bound TMPRSS2 proteases, the spike is cleaved and immediate plasma membrane fusion can occur [47]. In their absence, the SARS-CoV-2 can be internalised via clathrin-mediated and non-clathrin-mediated endocytosis, and the cleavage in the spike takes place in the endosome by endosomal cysteine proteases cathepsin B and L [48,49]. Because the ACE2 receptor is abundantly expressed in lung cells, a major target of SARS-CoV-2 infection, several hypotheses on how SARS-CoV-2 can disrupt the GPCR network in lung cells have been postulated. One of them considers the possibility that SARS-CoV-2 may hijack GPCR signalling pathways to dysregulate lung ion and fluid transport through the modulation of transepithelial transport processes, especially those involving the cystic fibrosis transmembrane conductance regulator (CFTR) and the epithelial Na^+^ channel (ENaC) [50]. Another hypothesis entertains the possibility that SARS-CoV-2 may activate the AT2 GPCR, which binds to ACE2 and activates the GRK/β-arrestin system, leading to enhanced clathrin-mediated endocytosis in lung cells [51]. However, there is so far little experimental proof supporting either of these hypotheses.

Our knowledge about the potential involvement of aGPCRs in infectious diseases is currently very limited. One of the main objectives of the still ongoing COST Action CA18240 Adher’nRise (Adhesion GPCR Network: Research and Implementation Set the path for future Exploration) is to explore the role of aGPCRs in bacterial and viral infectious diseases. To contribute to the meeting of this goal, we explore, in the present study, the potential role of aGPCRs in SARS-CoV-2 infection of Calu-3 and Caco-2 cells.

## 2. Materials and Methods

### 2.1. Cell Lines and Virus

Calu-3 cells (a cell line derived from lung adenocarcinoma, ATCC HTB-55, ATCC, Manassas, VA, USA) were maintained in Dulbecco’s Modified Eagle’s Medium (DMEM)—high glucose (4500 mg/L) with L-glutamine (4 mM), 10% fetal bovine serum (FBS), 100 U/mL penicillin and 100 µg/mL streptomycin. Caco-2 cells (a cell line derived from colorectal adenocarcinoma, HTB-37, ATCC) were maintained in DMEM—high glucose (4500 mg/L) with L-glutamine (4 mM), 20% FBS, 100 U/mL penicillin and 100 µg/mL streptomycin. Vero E6 (ECACC cat. no. 85020206, UK Health Security Agency, Salisbury, UK) were maintained in DMEM—high glucose (4500 mg/L) supplemented with L-glutamine (4 mM), 10% FBS, 100 U/mL penicillin and 100 µg/mL streptomycin (all Merck, Darmstadt, Germany). The cells were grown in a humidified incubator maintained at 37 °C with 5% CO_2_ and were passaged every 4−5 days (Calu-3 and Caco-2 cells) or 2−3 days (Vero E6). SARS-CoV-2 (strain hCoV-19/Czech Republic/NRL_6632_2/2020) was isolated in a biosafety level 3 laboratory from a nasopharyngeal swab by inoculating Vero CCL81 cells before being expanded by two additional passages in Vero CCL81 cells, as described [52]. Work with the SARS-CoV-2 was performed in the biosafety level 3 laboratory in accordance with the permit issued by the State Office for Nuclear Safety, Department of Non-Proliferation, Chemical and Biological Weapons Prohibition Division (permit number SÚJB/OKZCHBZ/6186/2020).

### 2.2. SARS-CoV-2 Preparation and Heat Inactivation

Isolated SARS-CoV-2 was subsequently passaged two times in Vero E6 cells in the same DMEM as above, but with reduced FBS (2%). Virus-containing supernatants were harvested, cleared by centrifugation (300× *g*, 5 min, 20 °C), filtered through 0.45 µm pore size cellulose-acetate filters (Merck, Darmstadt, Germany) and stored at −80 °C. Infectious titres were determined as the 50% tissue culture infectious dose (TCID50) by endpoint titration using serial 5-fold dilutions of SARS-CoV-2 in triplicate on Vero E6 cells according to the Reed and Muench method [53]. SARS-CoV-2 obtained from infected Vero E6 cells was directly used for infection of Calu-3 and Caco-2 cells (described in Section 2.8). For experiments with non-infectious viruses, SARS-CoV-2 was heat inactivated at 65 °C for 20 min. Virus-cleared conditioned media was prepared by centrifugation of SARS-CoV-2 obtained from infected Vero E6 cells through a cushion of 20% (*w*/*w*) sucrose in phosphate-buffered saline (PBS) (90,000× *g*, 3 h, 4 °C). The supernatant was filtered three times through 0.1-µm filters (Merck, Darmstadt, Germany).

### 2.3. Preparation of Lipid Nanoparticles

Lipid nanoparticles (LNPs) for the delivery of siRNA were prepared as described previously [54]. Briefly, for each siRNA sample and control, equal volumes of siRNA solution (53.3 ng siRNA/μL in 10 mM citrate buffer, pH 3.0) and 5 mM solution of lipids in ethanol (5 mM corresponds to the total concentration of the lipids) were mixed in a herringbone-type microfluidic chip. The ethanol phase contained a mixture of the XMAN5 lipid, 1,2-dioleoyl-sn-glycero-3-phosphoethanolamine (DOPE, TCI Europe, Haven, Belgium), cholesterol (Sigma−Aldrich, St. Louis, MO, USA) and 1,2-dimyristoyl-rac-glycero-3-methylpolyoxyethylene (DMG-PEG2000, NOF America Corp., White Palins, NY, USA) at a molar ratio of 22/33/43.5/1.5. After assembly, the obtained solution of LNPs was immediately diluted by the same volume of PBS and dialysed against PBS in a 10,000 MWCO cassette (Invitrogen, Carlsbad, CA, USA) at room temperature for 3 h. The average particle size was 83 ± 7 nm, and the entrapment efficiency of siRNA was over 80%.

### 2.4. Knockdown in Caco-2 and Calu-3 Cell Lines

LNPs containing commercially available siRNAs (Supplementary Table S1) were diluted with PBS to 5 ng/µL and added to freshly resuspended cells. For 48-well plates, 1 × 10^5^ Caco-2 cells or 1.4 × 10^5^ Calu-3 cells in 0.2 mL of the respective cultivation media were used. Immediately after seeding, 10 µL or 15 µL of LNPs were added to the Caco-2 or Calu-3 cells, respectively. For 96-well plates, 5 × 10^4^ Caco-2 cells or 7 × 10^4^ Calu-3 cells in 0.1 mL of the respective cultivation medium were used. Immediately after seeding, 5 µL of LNPs was added to the Caco-2 and Calu-3 cells. Knockdown efficiency was analysed 72 hrs after the first addition of LNPs (at the time of the infection with SARS-CoV-2 and the second addition of LNPs) and 24 and 48 hrs after the second addition of LNPs by RT-qPCR with primers specific to the selected aGPCRs.

### 2.5. Determination of LNP Cytotoxicity Using an XTT Assay

Cytotoxicity was evaluated in knockdown Calu-3 and Caco-2 cells treated as described above (i.e., by the addition of LNPs, followed by a second addition of LNPs after 3 days and viability measurement after two more days). Cell viability was determined in 96-well plates by adding a 50:1 mixture of an XTT labelling reagent (1 mg/mL) and a PMS electron-coupling reagent (0.383 mg/mL; both Merck, Darmstadt, Germany). The absorbance of the samples at 450 nm was measured after 4 h of incubation at 37 °C, 5% CO_2_ using an EnVision 2105 microplate reader (Perkin Elmer, Waltham, MA, USA).

### 2.6. RNA Isolation

Cells were harvested into 100 µL of TRIzol reagent (Thermo Fisher Scientific, Waltham, MA, USA), and RNA was isolated according to the manufacturer’s protocol and diluted to a concentration of 80 ng/μL.

### 2.7. RT-qPCR and mRNA Expression Analysis

Specific primers against 32 adhesion GPCRs and two internal control genes, GAPDH and peptidylprolyl isomerase A (PPIA), were designed using the NCBI software Primer-BLAST (https://www.ncbi.nlm.nih.gov/tools/primer-blast, last accessed on 29 March 2023). The accession number of each gene was obtained from the NCBI database (https://www.ncbi.nlm.nih.gov/nuccore, last accessed on 29 March 2023). The primer designing parameters were as follows: (i) PCR product size—70–130 bp; (ii) primer melting temperature—min 53 °C, opt 55 °C, max 58 °C; (iii) each primer was required to span the exon–exon junction. The primers were synthesized at Eurofins Genomics (Prague, Czech Republic) and are listed in Supplementary Table S2. The number of aGPCR mRNA copies was determined by reverse-transcription quantitative PCR (RT-qPCR) employing the Luna Universal Probe One-Step RT-qPCR Kit (New England Biolabs, Ipswich, MA, USA). In each reaction, 100 ng of RNA was used, and forward and reverse primers were then added to reach a final concentration of 0.4 μM. RT-qPCR cycles were performed as follows: 55 °C for 10 min for cDNA preparation, 95 °C for 1 min for initial denaturation, followed by 40 cycles of denaturation at 95 °C for 10 s, subsequent extension at 65 °C for 30 s and incubation at 72 °C for 1 min using a Bio-Rad real-time PCR cycler CFX Opus 96, and then being analysed using Bio-Rad CFX Maestro software (Bio-Rad, Hercules, CA, USA). Melting curve analysis followed each amplification for verification of the correct amplicon and absence of nonspecific products. Representative melting curves for four aGPCRs, which were selected for further analysis, are shown in Supplementary Figure S2C,D.

**Figure 1:**
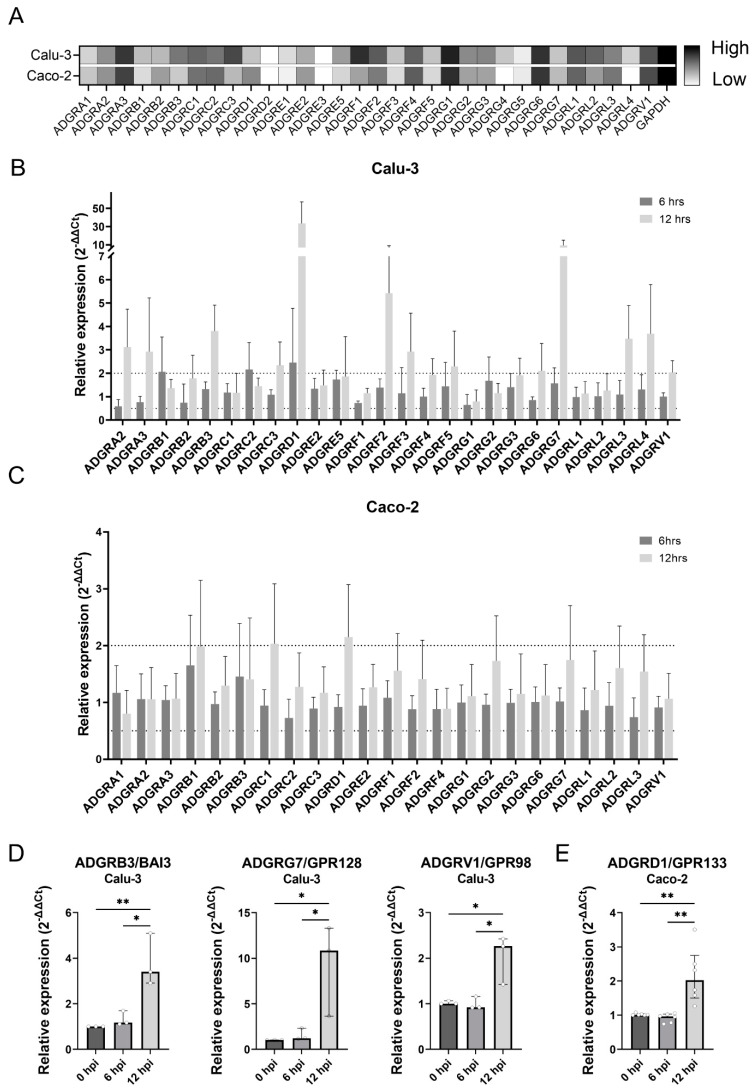
Infection with SARS-CoV-2 increased the mRNA expression of several aGPCRs. (**A**) Heatmap of relative mRNA expression of aGPCRs in cells of the lung adenocarcinoma cell line Calu-3 and of the colorectal adenocarcinoma cell line Caco-2. Lighter shades represent low relative expression (high ΔCt values) and darker shades stand for high relative expression (low ΔCt values). Relative expression changes in the aGPCRs in cells of the Calu-3 (**B**) and Caco-2 (**C**) cell lines upon infection with SARS-CoV-2. Calu-3 and Caco-2 cells were infected with SARS-CoV-2 with MOI of 2 and harvested 6 and 12 h post infection (h.p.i.). The values shown represent means of three (Calu-3 cells) and six (Caco-2 cells) independent biological replicates. Outliers were identified and eliminated based on the ROUT method (Q = 1%). aGPCRs with significantly altered relative expression upon infection with SARS-CoV-2 at an MOI of 2 in cells of the Calu-3 (**D**) and Caco-2 (**E**) cell lines according to ANOVA. The graphs present medians with the interquartile range from three (Calu-3 cells) and six (Caco-2 cells) independent biological replicates performed in technical duplicates. The relative expression of aGPCRs at 6 h.p.i. and 12 h.p.i. vs. 0 h.p.i. and 6 h.p.i. vs. 12 h.p.i. were compared by one-way ANOVA (*n* = 3 for Calu-3 cells and *n* = 6 for Caco-2 cells, * *p* < 0.05, ** *p* < 0.01) in GraphPad Prism 10.0.2.

**Figure 2:**
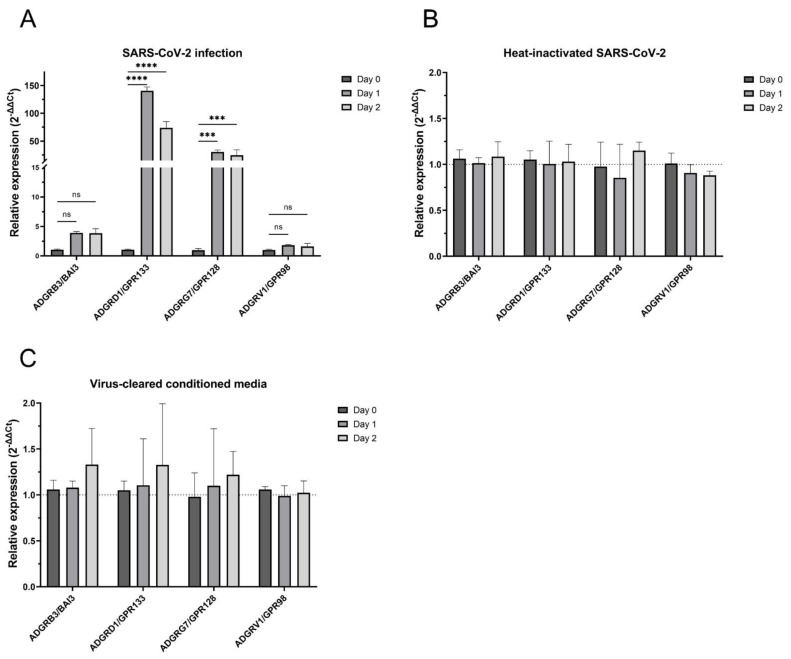
Replication-competent SARS-CoV-2, but not heat-inactivated SARS-CoV-2 or SARS-CoV-2-cleared conditioned media, increased the mRNA levels of aGPCRs in Calu-3 cells. Relative mRNA expression of selected aGPCRs in Calu-3 cells after (**A**) infection of SARS-CoV-2 with an MOI of 0.01 (the relative expression of aGPCRs at day 1 and day 2 vs. day 0 groups were compared by two-way ANOVA (*n* = 3, ns—not significant, *** *p* < 0.001, **** *p* < 0.0001)), (**B**) treatment with heat-inactivated SARS-CoV-2 and (**C**) treatment with virus-cleared conditioned media. Graphs represent means with standard deviations of three independent biological replicates.

**Figure 3:**
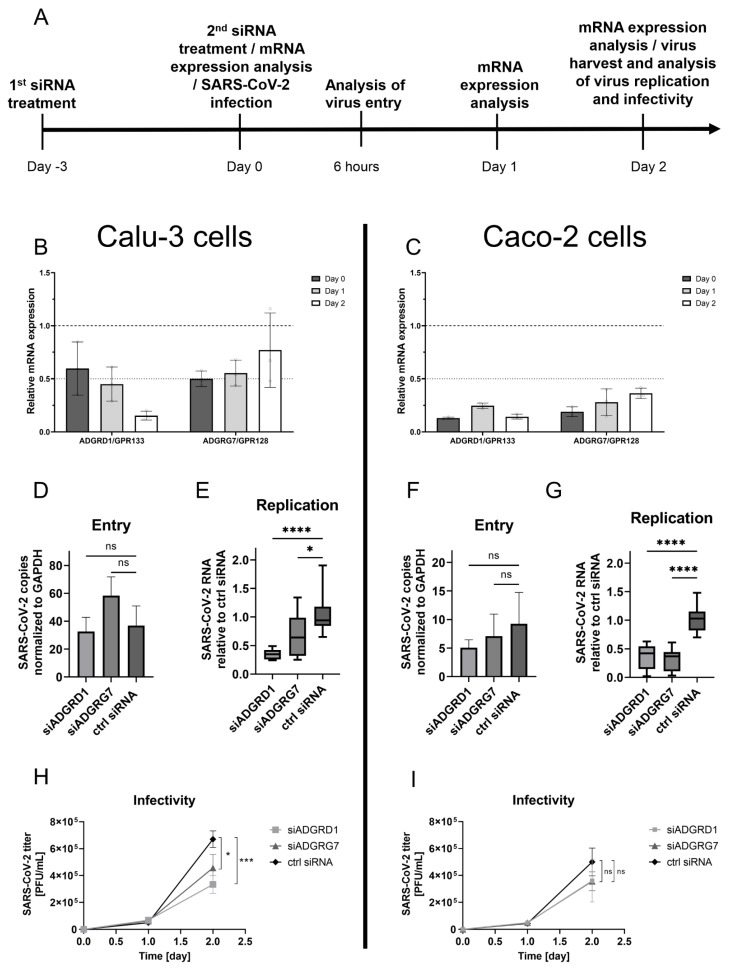
Knockdown of ADGRD1/GPR133 and ADGRG7/GPR128 reduced SARS-CoV-2 replication. (**A**) Experimental design. Downregulation of endogenous ADGRD1/GPR133 and ADGRG7/GPR128 mRNA expression in Calu-3 (**B**) and Caco-2 (**C**) cells after treatment with siRNA delivered by XMAN LNPs on day −3 and day 0 was estimated three days after the first siRNA transfection, and then on day 1 and day 2 after the second siRNA transfection by RT-qPCR. Graphs present means ± standard deviations of three independent biological replicates. The effect of downregulation of ADGRD1/GPR133 and ADGRG7/GPR128 mRNA expression on SARS-CoV-2 entry in Calu-3 (**D**) and Caco-2 (**F**) cells was determined after six hours post infection with SARS-CoV-2 at an MOI of 0.2. The SARS-CoV-2 copies normalised to GAPDH for siADGRD1 and siADGRG7 vs. ctrl siRNA were compared by one-way ANOVA (*n* = 3, ns—not significant). Total cellular RNA was isolated and SARS-CoV-2 RNA copies were quantified as specified in Methods. The effect of mRNA downregulation of ADGRD1/GPR133 and ADGRG7/GPR128 mRNA expression on SARS-CoV-2 replication in Calu-3 (**E**) and Caco-2 (**G**) cells was determined after two days post infection with SARS-CoV-2 at an MOI of 0.01. Media harvested from the cells were used to quantify the SARS-CoV-2 RNA from virions newly released from the cells by using RT-qPCR with SARS-CoV-2-specific primers. The SARS-CoV-2 RNA relative to ctrl siRNA for siADGRD1 and siADGRG7 vs. ctrl siRNA were compared by one-way ANOVA (*n* = 3, * *p* < 0.05, **** *p* < 0.0001). The effect of downregulation of ADGRD1/GPR133 and ADGRG7/GPR128 mRNA expression on SARS-CoV-2 infectivity in Calu-3 (**H**) and Caco-2 (**I**) cells was determined after one and two days post infection with SARS-CoV-2 at an MOI of 0.01. Media harvested from cells were analysed in a plaque assay with Vero E6 cells (Supplementary Figure S5 with representative images of plaque assay). The SARS-CoV-2 titres for siADGRD1 and siADGRG7 vs. ctrl siRNA were compared by one-way ANOVA (*n* = 3, ns—not significant, * *p* < 0.05, *** *p* < 0.001).

For heatmaps and experiments with SARS-CoV-2 infection at multiplicity of infection (MOI) of 2 with short time points (6 and 12 h post infection [h.p.i], Figure 1, Supplementary Figures S1 and S2), GAPDH was used as an internal control. In the rest of the experiments, the obtained Ct values of aGPCRs were normalised against the geometric mean of the Ct values for two internal controls, namely GAPDH and PPIA [55], resulting in ΔCt values (Figures 2 and 3B,C; Supplementary Figures S3 and S4D,E). GAPDH and PPIA were chosen based on their best expression stability within other broadly used internal controls, specifically upon SARS-CoV-1 infection [56]. In infection experiments with SARS-CoV-2, ΔCt values for infected samples were normalised against ΔCt values of uninfected samples from the same time point after infection. Expression fold change (exp) values were obtained using the formula exp = 2^−ΔΔCt^ where ΔΔCt stands for the difference between the ΔCt of infected samples and the ΔCt of uninfected samples. In knockdown experiments, siRNA-treated samples were normalised to control siRNA-treated samples harvested at the same time point. All data were generated in at least three biological replicates and analysed statistically by one-way ANOVA in GraphPad Prism 10.0.2 software (GraphPad, La Jolla, CA, USA). *p*-values < 0.05 were considered to be statistically significant. Values are presented as means ± the standard deviation.

### 2.8. Infection by SARS-CoV-2

For initial analysis of the mRNA expression of aGPCRs, Calu-3 and Caco-2 cells were seeded in a 75 cm^2^ flask the day before SARS-CoV-2 infection, infected with SARS-CoV-2 at a MOI of 2 and harvested 6 and 12 h after infection. Cells were harvested into 3 mL of TRIzol reagent (Thermo Fisher Scientific, Waltham, MA, USA).

For analysis of the aGPCR knockdown effect on SARS-CoV-2 infection, the cul-tivation media from the LNPs-treated cells was replaced by the media containing only 2% FBS prior to infection. SARS-CoV-2 was added at MOI of 0.01 to 48-well plates and at MOI of 0.2 to 96-well plates. The cells were incubated with the virus for 1 h at 37 °C in an atmosphere with 5% CO_2_; the unbound virus was then aspired before the cells were washed three times with PBS and supplemented with suitable cultivation media containing newly added LNPs (as described above).

### 2.9. ddPCR

SARS-CoV-2 entry into the cells was monitored by direct quantification of SARS-CoV-2 RNA copies. The LNPs-treated and SARS-CoV-2-infected cells from 96-well plates were lysed 6 h.p.i. in 100 µL of Trizol reagent (Thermo Fisher Scientific, Waltham, MA, USA), and the total RNA was then isolated according to the manufacturer’s protocol. Isolated RNA was diluted to 20 ng/µL and reversely transcribed using the Maxima first strand cDNA synthesis kit for RT-qPCR (Thermo Fisher Scientific, Waltham, MA, USA). For each reaction, 4 µL of the 5× reaction mix, 2 µL of the Max enzyme mix and 14 µL of the RNA sample were used. Samples were incubated for 10 min at 25 °C, 30 min at 50 °C and 5 min at 85 °C. Reversely transcribed cDNA was used as a template for the ddPCR. For the quantification of SARS-CoV-2 copies, FAM-labelled probes and primers from the E-Sarbeco kit (GeneriBiotech, Hradec Králové, Czech Republic) were used. For the quantification of GAPDH, the same primers as for RT-qPCR were used (Supplementary Table S2). The ddPCR reaction for SARS-CoV-2 quantification consisted of 20 µL of the mixture per well, containing 12 µL ddPCR Probe Supermix (no dUTP, Bio-Rad, Hercules, CA, USA), 6 µL E-Sarbeco primer mix (GeneriBiotech, Hradec Králové, Czech Republic) and 6 µL of reversely transcribed cDNA. For GAPDH quantification, the ddPCR reaction consisted of 20 µL per well of a mixture containing 11 μL of QX200 ddPCR EvaGreen Supermix (Bio-Rad, Hercules, CA, USA), 250 nM of primers and 6 µL of cDNA. The ddPCR reactions were incorporated into droplets using the QX100 Droplet Generator (Bio-Rad, Hercules, CA, USA). Nucleic acids were amplified with the following cycling conditions: 10 min at 95 °C, 45 cycles of 30 s at 95 °C and 59 °C for 60 s, with a final droplet cure step of 10 min at 98 °C using a Vapo.Protect Mastercycler (Eppendorf, Hamburg, Germany). The droplets were read and analysed using a Bio-Rad QX200 system and QuantaSoft software (version 1.7.4.0917) in ‘absolute quantification’ mode. Only wells containing ≥ 11,000 droplets were accepted for further analysis. Finally, SARS-CoV-2 RNA copies were normalised against the number of copies for GAPDH.

### 2.10. Plaque Assay

The LNPs-treated Caco-2 and Calu-3 cells were infected in triplicate with SARS-CoV-2. Supernatants from each triplicate were harvested at 24 and 48 h.p.i. and were titrated in 10-fold serial dilution (10 μL into 90 μL of media, four dilution steps in total, in monoplicate). The suspension was mixed with 1.25 × 10^5^ Vero E6 cells, and the 48-well plates were incubated for 4 h at 37 °C and 5% CO_2_. After the incubation, 3% carboxymethyl cellulose was added in a ratio of 1:1 into the wells. The plates were incubated for 5 days at 37 °C in an atmosphere containing 5% CO_2_. After the incubation period, the cultivation media and carboxymethyl cellulose were aspirated and the cells were washed with 1× PBS. The cells were fixed and stained with Naphtol black solution (1 g Naphtol Blue Black; 60 mL glacial acetic acid; 13.6 g sodium acetate trihydrate per 1 L distilled water; all Merck, Darmstadt, Germany) for 45 min at room temperature. After the incubation, the cells were washed with distilled water and air-dried. The plaques were manually counted and the virus yield was determined by calculating PFU per mL.

### 2.11. Quantification of SARS-CoV-2 RNA in the Media

For the quantification of SARS-CoV-2 RNA from virions newly released from the cells in the media 24 and 48 h.p.i., the DBdirect COVID-19 Multiplex RT-PCR kit (DIANA Biotechnologies, Vestec, Czech Republic) was used. Samples were prepared and analysed according to the manufacturer’s protocol, then modified for the 384-well format. Briefly, 0.75 μL of water and 1.25 μL of each Enhancer mix, Primer mix and Enzyme mix were used with 0.5 μL of the respective culture medium as a template on a 384-well plate. Nucleic acids were amplified with the following cycling conditions: 10 min at 50 °C, 2 min at 95 °C, 45 cycles of 5 s at 95 °C, 15 s at 60 °C and 15 s at 72 °C, followed by a final step at 40 °C for 30 s using a Vapo.Protect Mastercycler (Eppendorf, Darmstadt, Germany).

### 2.12. Adhesion GPCR Expression Analysis from Published Datasets

Adhesion GPCR expression analysis was performed using data published in the GSE213759 dataset of Bouhaddou et al. [57], the GSE148729 dataset of Wyler et al. [58] and the GSE252056 dataset of Mali et al. [59]. In these analyses, similar strains to our SARS-CoV-2 were used, namely ones that contained the D614G amino acid change in the spike protein. The data were processed as follows: raw read counts were normalised using the TPM (transcripts per kilobase million) method, transformed to Z-scores and visualised as a clustered heatmap. Differential gene expression analysis was performed using the DESeq2 package (Bioconductor). All the analyses were performed in R. The individual sample datasets used in the analyses are listed in Supplementary Table S3.

## 3. Results

### 3.1. Effect of SARS-CoV-2 Infection on aGPCRs’ mRNA Levels

In our broader effort to investigate the possible role of adhesion G protein-coupled receptors (aGPCRs) in the viral infection of mammalian cells, we initially determined the endogenous mRNA levels of aGPCRs in eight human cell lines commonly used in virus research. The expression profiles of all aGPCRs, with the exception of merely putative ADGRE4, were determined by RT-qPCR using primers spanning the exon–exon junction. We evaluated the relative expression levels of individual aGPCRs and identified those which were highly expressed across all tested cell lines, those with overall low expression and those with markedly variable expression levels among the cell lines tested (Figure 1A and Supplementary Figure S1). We focused on the lung adenocarcinoma cell line (Calu-3) and the colorectal adenocarcinoma cell line (Caco-2), which are widely used in SARS-CoV-2 infection experiments. In both cell lines, ADGRA3, ADGRG1 and ADGRG6 were highly expressed while ADGRD2 and ADGRE3 were almost absent; however, most aGPCRs showed a wide range of expression levels that are clearly distinct in several cases (Figure 1A).

To examine changes in the mRNA levels of individual aGPCRs during infection with SARS-CoV-2, we infected Calu-3 and Caco-2 cells with the virus at MOI of 2 and analysed the mRNA levels by RT-qPCR at 6 and 12 h post infection (Figure 1B,C). The high MOI allowed us to maximise the amount of cells infected, while analysis after a maximum of 12 h minimised the cytopathic effect of SARS-CoV-2. Only aGPCRs where reproducible amplification was achieved were included in Figure 1B,C. Our analyses of relative changes in the expressions of different aGPCRs upon SARS-CoV-2 infection revealed 17 and 6 aGPCRs with mRNA expressions trending upwards in Calu-3 and Caco-2 cells, respectively. Upon SARS-CoV-2 infection, three and one aGPCRs exhibited mRNA trending downwards in Calu-3 and Caco-2 cells, respectively. The changes were overall less pronounced in Caco-2 cells compared to Calu-3 cells. ANOVA identified only four changes in aGPCR mRNA expression as being statistically significant. In Calu-3 cells, the relative mRNA expression of ADGRB3/BAI3, ADGRG7/GPR128 and ADGRV1/GPR98 increased 3.4 times, 11 times and 2.3 times, respectively (Figure 1D). The mRNA level of ADGRD1/GPR133 was upregulated in both the cell lines tested, even though it only reached statistical significance in Caco-2 cells (Supplementary Figure S2A and Figure 1E). It was the only candidate reaching statistically significant changes in expression in Caco-2 cells. In contrast, the changes in mRNA expression of ADGRB3/BAI3, ADGRG7/GPR128 and ADGRV1/GPR98 upon SARS-CoV-2 infection were non-significant in cells of the Caco-2 cell line (Supplementary Figure S2B). All four candidate aGPCRs that showed a significant mRNA expression increase in Calu-3 or Caco-2 cells were selected for further analysis.

### 3.2. Only Replication-Competent SARS-CoV-2 Increased mRNA Levels of aGPCRs

To confirm that the increase in mRNA expression of GPCRs was caused by the infectious, replication-competent virus, we infected Calu-3 and Caco-2 cells with SARS-CoV-2 at a low MOI of 0.01. Furthermore, we treated the cells with heat-inactivated SARS-CoV-2 and with virus-cleared conditioned media. The mRNA expression of ADGRB3/BAI3, ADGRD1/GPR133, ADGRG7/GPR128 and ADGRV1/GPR98 was analysed prior to (as well as 24 h and 48 h after) being infected with SARS-CoV-2, as well as after treatment with heat-inactivated SARS-CoV-2 and virus-cleared conditioned media.

In Calu-3 cells, the relative mRNA expression upon infection with infectious SARS-CoV-2 exhibited a pronounced increase in the cases of ADGRD1/GPR133 and ADGRG7/GPR128, a small increase in the case of ADGRB3/BAI3 and a negligible change in that of ADGRV1/GPR98 mRNA levels after 24 and 48 h (Figure 2A). The increase in mRNA expression of ADGRD1/GPR133 and ADGRG7/GPR128 after 24/48 h increased more than three times compared to the mRNA expression change 12 h after infection (Figure 1D and Supplementary Figure S2A). Interestingly, the change in mRNA expression for ADGRB3/BAI3 and ADGRV1/GPR98 did not exhibit any such effect. Instead, the mRNA expression changes remained similar to after 12 h post infection. The treatment with heat-inactivated SARS-CoV-2 (Figure 2B) or virus-cleared conditioned media (Figure 2C) resulted in negligible changes in the mRNA expression of all four aGPCRs.

In Caco-2 cells (Supplementary Figure S3A), no increase in mRNA expression of ADGRB3/BAI3, ADGRG7/GPR128 and ADGRV1/GPR98 was detected even 48 h after infection, thus confirming our results after 12 h post infection (Supplementary Figure S2B). It is worthy of note that the increase in mRNA expression of ADGRD1/GPR133 observed 12 h after infection was not sustained after 24 or 48 h. Changes in the mRNA levels of the aGPCRs under study in Caco-2 cells treated with heat-inactivated SARS-CoV-2 or virus-cleared conditioned media were merely subtle (Supplementary Figure S3B,C).

### 3.3. Knockdown of ADGRD1/GPR133 and ADGRG7/GPR128 Reduced SARS-CoV-2 Replication

To further explore the role of ADGRD1/GPR133 and ADGRG7/GPR128 during SARS-CoV-2 infection, we assessed the effect of the siRNA downregulation of aGPCRs on SARS-CoV-2 entry, replication and infectivity. Both epithelial cell lines used, Calu-3 and Caco-2, are fairly resistant to transfection, which makes the use of siRNA challenging. To achieve effective transfection, we used lipid nanoparticles (LNPs) based on recently described adamantane-based lipidoids XMANs [54]. These particles have proven to be capable of delivering siRNA to cell lines that are difficult to transfect, including primary cells.

The cells were treated with aGPCR-specific siRNA three days before SARS-CoV-2 infection and then again on the day of infection (Figure 3A). This treatment did not affect the cell viability (Supplementary Figure S4A,B) or cell morphology (Supplementary Figure S4C). In both Calu-3 and Caco-2 cell lines, sufficient downregulation was achieved only for ADGRD1/GPR133 and ADGRG7/GPR128 (Figure 3B,C). In the cases of ADGRB3/BAI3 and ADGRV1/GPR98, we observed only weak downregulation (Supplementary Figure S4D,E), so we excluded these two aGPCRs from subsequent analyses.

To address the possibility that aGPCRs can influence the entry of SARS-CoV-2, we analysed the effectiveness of SARS-CoV-2 entry into Calu-3 and Caco-2 cells after the siRNA downregulation of ADGRD1/GPR133 and ADGRG7/GPR128. The number of SARS-CoV-2 copies in both cell lines was determined 6 h after infection in lysed cells using droplet digital PCR. The number of SARS-CoV-2 copies in cells of both cell lines (Figure 3D,F) did not differ significantly from that in cells treated with control siRNA, indicating that downregulation of ADGRD1/GPR133 and ADGRG7/GPR128 has no or minimal effect on SARS-CoV-2 entry.

Because we did not observe any effect of aGPCR downregulation in regard to SARS-CoV-2 entry, we analysed the amount of newly released virus in the supernatant as an indication of SARS-CoV-2 replication. The quantification was performed 48 h after infection by RT-qPCR with SARS-CoV-2-specific primers. These experiments showed that, in both Calu-3 and Caco-2 cells, the downregulation of ADGRD1/GPR133 and ADGRG7/GPR128 led to a statistically significant, more than two-fold decrease in the number of released virus particles (Figure 3E,G). Next, we used a plaque assay that allows for the direct detection of viable infectious virus to reveal possible subtle defects in the virus’s infectivity caused by the downregulation of aGPCRs. The cultivation media used with aGPCR-downregulated SARS-CoV-2-infected cells were collected one day and two days after infection, and the virus yield was then determined by a plaque assay in Vero E6 cells. SARS-CoV-2 harvested at 48 h after infection from aGPCR-downregulated Calu-3 showed significantly reduced viral titres compared to the titre of SARS-CoV-2 in control siRNA-downregulated cells (Figure 3H). The reduction in SARS-CoV-2 titre was more distinctive in ADGRD1/GPR133-downregulated Calu-3 cells than in ADGRG7/GPR128-downregulated cells. On the other hand, a smaller, non-significant reduction in the virus titre was observed in aGPCR-downregulated Caco-2 cells compared to control siRNA-downregulated cells (Figure 3I).

### 3.4. Reanalysis of aGPCR Expression Levels after SARS-CoV-2 Infection Based on Published mRNA Datasets

To compare our aGPCR mRNA expression analysis with published SARS-CoV-2 RNAseq analyses, we selected several datasets where the experimental conditions for SARS-CoV-2 infection were similar to the ones used in our experiments. In this comparison we included the previously published RNAseq datasets GSE148729 (Wyler et al. [58]), GSE213759 (Bouhaddou et al. [57]) and GSE252056 (Mali et al. [59]). In the acquisition of all three datasets, Calu-3 cells were infected with SARS-CoV-2, which was isolated early during the COVID-19 pandemic, using similar amounts of virus and a similar duration of infection, albeit not identical to the ones used in our experiments. We also considered additional datasets, but they either did not contain raw read counts and were incompatible with our pipeline (as with, for example, GSE197521 and GSE151513) or they consisted of data on cells pretreated with an infection-enhancing compound (as in the case of dataset GSE236942). We constructed heatmaps comparing normalised aGPCR expression levels between mock- and SARS-CoV-2-infected Calu-3 cells (Supplementary Figures S6–S8) as well as mock- and SARS-CoV-2-infected Caco-2 cells (for dataset GSE148729) (Supplementary Figure S9). Our heatmaps revealed very large variability both among the datasets and replicates of the same experiments. Moreover, this variability was reflected also in graphs showing the relative gene expression of aGPCRs in Calu-3 (Supplementary Figures S10–S12) and Caco-2 cells (Supplementary Figure S13) after infection with SARS-CoV-2.

**Figure 4:**
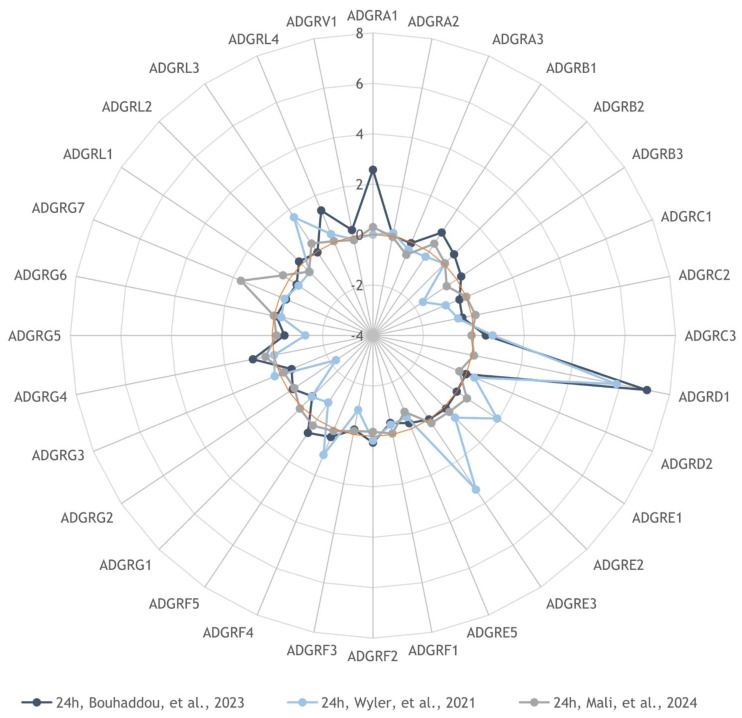
Summary analysis of relative gene expression of aGPCRs in Calu-3 cells after SARS-CoV-2 infection based on previously published mRNA datasets. The radar plot displays the relative gene expression of aGPCRs in Calu-3 cells 24 h after infection, while the size of the circle indicates log_2_ values (fold change). Dark blue circles, GSE213759; light blue circles, GSE148729; and grey circles, GSE252056. The light brown circle represents a zero-fold change.

Our comparison of the relative gene expression of aGPCRs in Calu-3 cells 24 h after infection is summarised as a radar plot in Figure 4. The overall agreement regarding the overexpression of aGPCRs across all three datasets is low. Two of the datasets indicate very high statistically significant upregulation of the ADGRD1 gene (58- and 135-fold) and small but significant upregulation of ADGRC3 (1.7- and 1.4-fold) and ADGRF4 (2.2- and 1.3-fold). The results from the last datasets (Mali et al.), however, do not corroborate these observations, showing only a small upregulation of ADGRF2 (1.5-fold) and very small upregulation of ADGRC1 (1.2-fold) 48 h after infection, which are, however, still statistically significant (Supplementary Figure S11). Only the datasets from Wyler et al. contain raw data from infected Caco-2 cells where overall smaller changes in aGPCR expression in comparison with Calu-3 cells were observed. Significant upregulation of aGPCR expression in Caco-2 cells was observed only for ADGRG6, ADGRE5, and ADGRA3, but overall the changes were very small (less than 1.3-fold). In summary, data regarding aGPCR gene expression from two datasets confirm our results through showing very high overexpression of ADGRD1 mRNA; however, they do not support the increase in ADGRG7 mRNA observed by us.

## 4. Discussion

Adhesion GPCRs are a class of enigmatic surface receptors with mostly unknown function, but with the amount of recent research progress, they might soon represent attractive therapeutic targets similar to many members of other GPCR families. Here we present data indicating that aGPCRs can play a role during SARS-CoV-2 infection of mammalian cells. We initially established the endogenous mRNA expression levels of 32 aGPCRs in cells of the lung adenocarcinoma cell line, Calu-3, and the colorectal adenocarcinoma cell line, Caco-2, with both these cell lines being commonly employed in SARS-CoV-2 studies, which allowed us to select aGPCRs in Calu-3 and Caco-2 cells with reasonably high basal mRNA expression for further experiments in the context of SARS-CoV-2 infection. Following SARS-CoV-2 infection of both cell lines, we identified ADGRB3/BAI3, ADGRV1/GPR98 and ADGRG7/GPR128 with increased mRNA expression in Calu-3 cells and ADGRD1/GPR133 with increased mRNA expression in Caco-2 cells. The ADGRD1/GPR133 also exhibited the highest mRNA expression increase in two out of three biological replicates in Calu-3 cells (over 45 times, Supplementary Figure S2A). Additional experiments using a lower MOI confirmed that the greatest mRNA expression increase in Calu-3 cells indeed concerned ADGRD1/GPR133, followed by ADGRG7/GPR128.

We re-analysed aGPCR expression data from the previously published RNAseq datasets GSE213759 [57], GSE148729 [58] and GSE252056 [59], during the acquisition of which Calu-3 cells were infected under similar conditions and with a similar SARS-CoV-2 strain isolated in the spring of 2020, as in our experiments. Although this comparison revealed large inter- and intra-experiment variability in the heatmaps, two of the RNAseq analyses confirmed the high relative fold increase in ADGRD1/GPR133 expression in Calu-3 cells 24 h after SARS-CoV-2 infection. On the other hand, no increase in ADGRG7/GPR128 expression was observed in these analyses. Nevertheless, examination of the GSE252056 dataset (Supplementary Figure S11) revealed 3.2-fold upregulation of ADGRG7 24 h post infection and striking 18.7-fold downregulation after 48 h of SARS-CoV-2 infection. However, both these changes lacked statistical significance. Similar abrupt changes have been observed also in other aGPCR expression levels during SARS-CoV-2 infection, which might explain the large variability among biological replicates observed both in our data and in the previously published datasets that we examined. Similarly to our own results, our re-analysis of data on SARS-CoV-2-infected Caco-2 cells from the GSE148729 dataset revealed less dramatic changes in aGPCR mRNA levels in comparison with results obtained with Calu-3 cells. Only four changes in aGPCR mRNA levels were significant, while the final fold changes were negligible. The transcriptomic profiling of SARS-CoV-2-infected Calu-3 and Caco-2 cells by Wyler et al. indeed found distinct expression patterns between the two cell lines [58]. Although both cell lines exhibited upregulation in genes, typically activated in response to ER stress and MAP kinase activation, no induction of the expression of genes regulated by interferon regulatory factors was observed in the Caco-2 cell line [58]. Interestingly, arrestin-related domain-containing protein-3 (ARRDC3) was significantly upregulated upon infection with SARS-CoV-2 in both cell lines. The ARRDC3 protein is known to mediate G-protein-coupled receptor signalling. In addition, several members of the chemokine family of GPCRs have been observed to be upregulated in Calu-3 cells upon infection, including, among others, CXCL10, CXCL11 and CCL2 [58].

The function of ADGRG7/GPR128 is mostly unknown. It is, however, known to be connected with different types of tumours, including endometrial carcinoma [36], breast carcinoma [38] and head and neck squamous cell carcinoma [37]. A mouse ADGRG7/GPR128 gene knockout model has established the importance of this aGPCR for the correct functioning of the intestines [60]. Although lung cells are the main target of SARS-CoV-2 infection, many reports have demonstrated that the gastrointestinal tract is also critically affected by SARS-CoV-2 infection (reviewed in [61]).

ADGRD1/GPR133 has been implicated as a critical regulator of the response to hypoxia and tumour growth in glioblastoma [29,30]. Recently, it has been found in the lung that there is high expression of ADGRD1/GPR133 and the transmembrane protein PTK7 at the whole-tissue level; however, individual cells preferentially express only ADGRD1/GPR133 or PTK7 rather than co-expressing both genes [30]. In this context, it is important to note that early hypoxia is a determinant of the progression of COVID-19 to its severe and critical stages [62,63]. Moreover, the transcription of ADGRD1/GPR133 is upregulated in hypoxic regions by hypoxia-inducible factor 1 α (Hif1α) [64], the same factor that has recently been shown to be activated in the lungs of adult Syrian hamsters infected with SARS-CoV-2 [65].

Furthermore, our results show that increased mRNA expression of ADGRD1/GPR133 is caused by replication-competent SARS-CoV-2. Neither heat-inactivated SARS-CoV-2 nor virus-cleared conditioned media increased the mRNA expression of ADGRD1/GPR133 and ADGRG7/GPR128. Finally, we have confirmed the interplay between the SARS-CoV-2 and the aGPCRs tested by infecting Calu-3 cells with siRNA-downregulated expression of ADGRD1/GPR133 and ADGRG7/GPR128. In such cells, we observed reduced SARS-CoV-2 replication and lower infectivity compared to Calu-3 cells treated with control siRNA. We have also detected reduced SARS-CoV-2 replication in Caco-2 cells with downregulated ADGRD1/GPR133 and ADGRG7/GPR128. However, the effect on infectivity was insignificant, probably due to the overall lower infectability of Caco-2 cells by SARS-CoV-2, as evidenced by the six to eight times lower amount of SARS-CoV-2 copies 6 h.p.i. in Caco-2 cells (Figure 3F) compared to Calu-3 cells (Figure 3D). Downregulation of ADGRD1/GPR133 and ADGRG7/GPR128 did not cause cytotoxicity or obvious changes in the morphology of Calu-3 and Caco-2 cells; however, we cannot exclude the possibility that aGPCR downregulation affected cell polarity. Because coronaviruses are efficiently transmitted in polarised cells (reviewed in [66]), the observed reduction in SARS-CoV-2 replication and infectivity could have been caused by a change in cell polarity.

## 5. Limitations of the Study

Our aGPCR mRNA analysis agreed with published RNAseq data only in the case of ADGRD1/GPR133, where similar upregulation was observed. However, neither of the three RNAseq analyses showed any statistically significant increase in ADGRG7/GPR128 upon SARS-CoV-2 infection. Two RNAseq studies showed small significant increases in ADGRC3 and ADGRF4 in Calu-3 cells 24 h post infection that we did not observe in our experiments after 12 h after infection. In addition, so far we have not been able to confirm, using commercially available antibodies, aGPCR expression changes in Calu-3 cells at the protein level, which constitutes a substantial limitation of our results. The biggest problem was posed by non-specific antibodies and our inability to overexpress aGPCRs in Calu-3 cells. We have therefore not been able to establish any direct or indirect interaction between aGPCR and SARS-CoV-2 at the protein level. In the future, we plan to employ ribosomal profiling if we are not able to confirm increased expression at the mRNA level by the Western blot method. We intend to analyse the effect of SARS-CoV-2 infection on aGPCR activation and downstream signalling. Additionally, we will utilise confocal microscopy to check for changes in the localisation of aGPCRs upon infection. If the levels of endogenous aGPCRs turn out to be too low, we will employ overexpression using lentiviral vectors. Furthermore, the reduced SARS-CoV-2 replication and infectivity in siRNA-downregulated Calu-3 cells needs to be confirmed using additional siRNA. Finally, another considerable limitation of our study is the use of two immortalised cell lines, the aGPCR profiles of which could be altered. Our findings therefore need to be confirmed in primary cells, such as primary airway cells and lung organoids.

## 6. Conclusions

Here, we present the first evidence that aGPCRs may play a role during SARS-CoV-2 infection of lung adenocarcinoma Calu-3 cells. We observed elevated mRNA expression of ADGRD1/GPR133 and ADGRG7/GPR128 in SARS-CoV-2-infected Calu-3 cells and slightly increased mRNA expression of ADGRD1/GPR133 in high MOI-infected Caco-2 cells. In addition, we detected lower SARS-CoV-2 replication and infectivity in Calu-3 cells with downregulated expressions of ADGRD1/GPR133 and ADGRG7/GPR128. In light of reanalysed RNAseq data and our findings, we believe that further research into the role of ADGRD1/GPR133 (but likely not ADGRG7/GPR128) in viral infections is warranted.

## Data Availability

Data is contained within the article and Supplementary Materials.

## Supplementary Materials

The following supporting information can be downloaded at: https://www.mdpi.com/article/10.3390/cells13100791/s1, Figure S1: Heatmap of relative mRNA expression of aGPCRs in six different cell lines; Figure S2: Relative mRNA expression of selected aGPCRs and representative melting curve graphs; Figure S3: Insignificant changes of relative mRNA expression of selected aGPCRs in Caco-2 cells after infection with SARS-CoV-2, exposure of heat-inactivated SARS-CoV-2 and virus-cleared conditioning media; Figure S4: Cytotoxicity of used siRNAs; Figure S5: Representative figures of plaque assays at 48 h.p.i.; Figures S6–S8: Comparison of adhesion GPCR expression in non-infected Calu-3 cells and cells after infection with SARS-CoV-2; Figure S9: Comparison of adhesion GPCR expression in non-infected Caco-2 cells and cells after infection with SARS-CoV-2; Figures S10–S12: Relative gene expression of adhesion GPCRs in Calu-3 cells after infection with SARS-CoV-2; Figure S13: Relative gene expression of adhesion GPCRs in Caco-2 cells after infection with SARS-CoV-2; Table S1: List of siRNA used for knockdown experiments; Table S2: Primers sequences used for RT-qPCR; Table S3: List of evaluated GEO samples.
