# Supplementary material for: Upregulation of mRNA Expression of ADGRD1/GPR133 and ADGRG7/GPR128 in SARS-CoV-2-Infected Lung Adenocarcinoma Calu-3 Cells
Source: Cells. 2024 May 7;13(10):791. doi: 10.3390/cells13100791 (PMC11119037; doi:10.3390/cells13100791)
Supplement: Supplementary file 1 [file cells-13-00791-s001.zip › cells-2958585-supplementary.pdf]

Supplementary Table S1. List of siRNAs used for knockdown experiments

Gene name	sense (5′->3′)	antisense (5′->3′)
ADGRB3 (BAI3)	GCGACCACCCAAAGAAGAAtt	UUCUUCUUUGGGUGGUCGCtt
ADGRD1 (GPR133)	GAGAUAUUGUGGAAGGGAAtt	UUCCCUUCCACAAUAUCUCca
ADGRG7 (GPR128)	GCGAGUGGUUGGACAGAUAtt	UAUCUGUCCAACCACUCGCgt
ADGRV1 (GPR98)	GAAACACGGGAGUACUAGAtt	UCUAGUACUCCCGUGUUUCca

All siRNAs used for aGPCRs downregulation were purchased from Thermo Fisher Scientific as Silencer Select Pre-designed siRNA products. Control siRNA (Silencer Negative Control #1 siRNA) was purchased from Applied Biosystems.

Supplementary Table S2. Primer sequences used for RT-qPCR

**Gene Name**	**GeneBank Access. No.**	**Primer Sequence (5′→3′)**
ADGRL1 (LPHN1)	NM_001008701.3	**F**-GGACAACATTAAATCCTGGG<br>**R**-GAAGGTGGTGAAGAGATAGG
ADGRL2 (LPHN2)	NM_012302.5	**F**-CCTGGAGACGGGATATGAA<br>**R**-GCAACCAGAAGACACCATTA
ADGRL3 (LPHN3)	NM_001322402.3	**F**-TCCGTAGAATGTGGAATGAC<br>**R**-AGAAGCCCCTCTCTGTTG
ADGRL4 (ELTD1)	NM_022159.4	**F**-CTCAAACCCACCCACATTAT<br>**R**-CCTATCTGTGACCTTTCGAT
ADGRE1 (EMR1)	NM_001974.5	**F**-TGCGATGCAAAGATATTGAT<br>**R**-TGGGAGAAGAGAAACCATCT
ADGRE2 (EMR2)	NM_013447.4	**F**-CACAATAGGCACCAGAGAC<br>**R**-GATCCTCCTCCTGCACAT
ADGRE3 (EMR3)	NM_032571.5	**F**-GGAAAGGTGGTTGGCTTAC<br>**R**-GCAGAGGCCTGGAAGA
ADGRE5 (CD97)	NM_001784.5	**F**-GGGACAGCCTGCACA<br>**R**-AGACACAGAATGCGAGAAAG
ADGRA1 (GPR123)	NM_001083909.3	**F**-CACCTGATCGCCTCCC<br>**R**-ACTGTCTTCAGATCCATGAGC
ADGRA2 (GPR124)	NM_032777.10	**F**-AGTCCTATGCTCCGGTTCTAT<br>**R**-CTGTGGTCCCGGTAGTT
ADGRA3 (GPR125)	NM_145290.4	**F**-CCCAGATACTCTGCCCAA<br>**R**-TTTCGGAGGTCCAATCTTTC
ADGRC1 (CELSR1)	NM_001378328.1	**F**-CGCCATCACAGTCAAGAG<br>**R**-TTCATTACTGCCTTCAGAGT
ADGRC2 (CELSR2)	NM_001407.3	**F**-GCCCAACATTGTCATCTCC<br>**R**-AGGCAGAATGACTGTTGTC
ADGRC3 (CELSR3)	NM_001407.3	**F**-TTACTTCTTTGGTGTGGAGG<br>**R**-TCCTTCATTGTGAACTCAGG
ADGRD1 (GPR133)	NM_198827.5	**F**-CAGGATACAACTGGAGATATTG<br>**R**-TGTACCTGCCGTAATAGAGA
ADGRD2 (GPR144)	NM_001395425.1	**F**-GACCTATGGCCCTAGAACT<br>**R**-CACTCTCTAGAAACCTGAAGC
ADGRF1 (GPR110)	NM_153840.4	**F**-AATGAAAGTTGGAGTGCTGT<br>**R**-ATGCCATCATTTTTCCCCA

ADGRF2 (GPR111)	NM_153839.7	**F**-TATCTTCTCCCTGCTCAATG<br>**R**-TTAAGGCTTCTCTTATCTTTGGA
ADGRF3 (GPR113)	NM_001321971.2	**F**-CCCGAGAAGGGACAGAG<br>**R**-ATGTACATAGACGGAGACCA
ADGRF4 (GPR115)	NM_153838.5	**F**-GATGGTGAGGCATCATGCTA<br>**R**-ATGAGGATCTTCACGGCA
ADGRF5 (GPR116)	NM_015234.5	**F**-TCTGGCTGTGTGATTGAATG<br>**R**-TTTGAGTTGGTTGGCCTTT
ADGRB1 (BAI1)	NM_001702.3	**F**-CTGGTTGTGGCCATTTCT<br>**R**-AGAGCCAGCAGTAGTTCAT
ADGRB2 (BAI2)	NM_001294335.2	**F**-CCCTCTCAGTAAAGCAAGATT<br>**R**-GTCATCCTATGTCCCTTGC
ADGRB3 (BAI3)	NM_001704.3	**F**-AACAACTTACAGAGGCCAAA<br>**R**-GTCTTGGGCAGCATTAAATC
ADGRG1 (GPR56)	NM_005682.7	**F**-GCCTTGATCTTCTTCTCCTT<br>**R**-ATGAAGATGAGGAAGCCTTG
ADGRG2 (GPR64)	NM_001079858.3	**F**-GTAACATCCCTGGAAGAAGA<br>**R**-ACATCATTGAGGCTTGTTGT
ADGRG3 (GPR97)	NM_170776.5	**F**-GAGCAACAACATGTACGAC<br>**R**-TAGTTTAGCCAGTATCTCTGC
ADGRG4 (GPR112)	NM_153834.4	**F**-GCCCTCCCAGGCTGT<br>**R**-ACTCCACTGCTTCTTCTTTC
ADGRG5 (GPR114)	NM_153837.4	**F**-AGTTTGTGCTTGGCAATCT<br>**R**-CTCCGGCCCTGAGAC
ADGRG6 (GPR126)	NM_020455.6	**F**-CAGCTATATTGGGTGTGGAA<br>**R**-CCCTTCGCAATTTCTCAAAA
ADGRG7 (GPR128)	XM_011513245.2	**F**-TTACTTTTGCCAGAATCCCA<br>**R**-TGACATCCTTTACCATTTGGA
ADGRV1 (VLGR1)	NM_032119.4	**F**-CTTTTCTTTCCTGAGGGGTC<br>**R**-GTGTCCTGACATCATACAGA
GAPDH	NM_002046.7	**F**-AGGTCGGAGTCAACGGA<br>**R**-AACATGTAAACCATGTAGTTGAG
PPIA	NM_001300981.2	**F**-CGCGTCTCCTTTGAGGTAA<br>**R**-GCTGTTCTTTTAGTCACTTGT

Supplementary Table S3. List of evaluated GEO samples

GSE148729

GEO ID	Sample ID
GSM4477876	Caco2_polyA-mock-24h-A
GSM4477877	Caco2_polyA-mock-24h-B
GSM4477878	Caco2_polyA-mock-4h-A
GSM4477879	Caco2_polyA-mock-4h-B
GSM4477886	Caco2_polyA-S2-12h-A
GSM4477887	Caco2_polyA-S2-12h-B
GSM4477888	Caco2_polyA-S2-24h-A
GSM4477889	Caco2_polyA-S2-24h-B
GSM4477890	Caco2_polyA-S2-4h-A
GSM4477891	Caco2_polyA-S2-4h-B
GSM4477892	Caco2_polyA-untr-4h-A
GSM4477893	Caco2_polyA-untr-4h-B
GSM4477894	Calu3_polyA_series1-untr-4h-A
GSM4477895	Calu3_polyA_series1-untr-4h-B
GSM4477896	Calu3_polyA_series1-mock-4h-A
GSM4477897	Calu3_polyA_series1-mock-4h-B
GSM4477898	Calu3_polyA_series1-mock-24h-A
GSM4477899	Calu3_polyA_series1-mock-24h-B
GSM4477906	Calu3_polyA_series1-S2-4h-A
GSM4477907	Calu3_polyA_series1-S2-4h-B
GSM4477908	Calu3_polyA_series1-S2-12h-A
GSM4477909	Calu3_polyA_series1-S2-12h-B
GSM4477910	Calu3_polyA_series1-S2-24h-A
GSM4477911	Calu3_polyA_series1-S2-24h-B
GSM4477916	Calu3_polyA_series2-12h-mock-1
GSM4477917	Calu3_polyA_series2-12h-mock-2
GSM4477920	Calu3_polyA_series2-12h-S2-1
GSM4477921	Calu3_polyA_series2-12h-S2-2
GSM4477922	Calu3_polyA_series2-4h-mock-1
GSM4477923	Calu3_polyA_series2-4h-mock-2
GSM4477926	Calu3_polyA_series2-4h-S2-1

GSE252056

GEO ID	Sample ID
GSM7993158	in3126_1_calu3_mock_24hrs
GSM7993159	in3126_2_calu3_mock_24hrs
GSM7993160	in3126_3_calu3_mock_24hrs
GSM7993161	in3126_4_calu3_sars_24hrs
GSM7993162	in3126_5_calu3_sars_24hrs
GSM7993163	in3126_6_calu3_sars_24hrs
GSM7993164	in3126_25_calu3_mock_48hrs
GSM7993165	in3127_1_calu3_mock_48hrs
GSM7993166	in3127_2_calu3_mock_48hrs
GSM7993167	in3127_3_calu3_sars_48hrs
GSM7993168	in3127_4_calu3_sars_48hrs
GSM7993169	in3127_5_calu3_sars_48hrs

GSE213759

GEO ID	Sample ID
GSM6593793	UK8_Calu3_Mock_10h_1
GSM6593794	UK8_Calu3_Mock_10h_2
GSM6593795	UK8_Calu3_Mock_10h_3
GSM6593796	UK8_Calu3_IC19_10h_1
GSM6593797	UK8_Calu3_IC19_10h_2
GSM6593798	UK8_Calu3_IC19_10h_3
GSM6593817	UK8_Calu3_Mock_24h_1
GSM6593818	UK8_Calu3_Mock_24h_2
GSM6593819	UK8_Calu3_Mock_24h_3
GSM6593820	UK8_Calu3_IC19_24h_1
GSM6593821	UK8_Calu3_IC19_24h_2
GSM6593822	UK8_Calu3_IC19_24h_3
GSM6593841	UK8_Calu3_Mock_48h_1
GSM6593842	UK8_Calu3_Mock_48h_2
GSM6593843	UK8_Calu3_Mock_48h_3
GSM6593844	UK8_Calu3_IC19_48h_1

GSM4477927	Calu3_polyA_series2-4h-S2-2	GSM6593845	UK8_Calu3_IC19_48h_2
GSM4477930	Calu3_polyA_series2-8h-S2-1	GSM6593846	UK8_Calu3_IC19_48h_3
GSM4477931	Calu3_polyA_series2-8h-S2-2		

Supplementary Methods

Cells

The human lung carcinoma cell line A549 (ATCC CCL-185), the human embryonic kidney HEK293T cell line (ATCC CRL-3216) and HeLa-derived TZM-bl cell line (NIH AIDS Regent Program, Division of AIDS, NIAID, NIH from Dr. John C. Kappese a Dr. Xiaoyun Wu) were maintained in Dulbecco's Modified Eagle's Medium (DMEM) high glucose(4500 mg/L) with L-glutamine (4 mM), 10% FBS, 100 U/mL penicillin and 100 μg/mL streptomycin. The human $CD4^+$ T-cell line MT4 (NIH AIDS Reagent Program, Division of AIDS, NIAID, NIH from Dr. Douglas Richman) and Jurkat clone E6-1 (ATCC, TIB-152) were maintained in RPMI 1640 w/o L-Glutamine and 25 mM Hepes supplemented with 10% FBS, penicillin (100 U/mL) and streptomycin (100 μg/mL). HepG2-NTCP (gift of Dr. Stephan Urban, Heidelberg University Hospital, Heidelberg, Germany) were cultivated in DMEM high glucose (4500 mg/L) with L-glutamine (4 mM), 10% FBS, 100 U/mL penicillin, 100 μg/mL streptomycin and puromycin (2 μg/mL) (all Merck, Germany). All cell lines were cultured at 37 °C under a humidified (>90%) atmosphere of 5% CO_2/95% air. All adherent cell lines were grown in 75-cm^2 flasks until reaching confluency and then were harvested into 3 mL of TRIzol reagent (Thermo Fisher Scientific, USA). For suspension cells (namely MT4 and Jurkat E6-1) was harvested 20×10^6 cells and lysed in 3 mL of TRIzol reagent (Thermo Fisher Scientific, USA). RNA was isolated according to the manufacturer's protocol and diluted to concentration of 80 ng/μL.

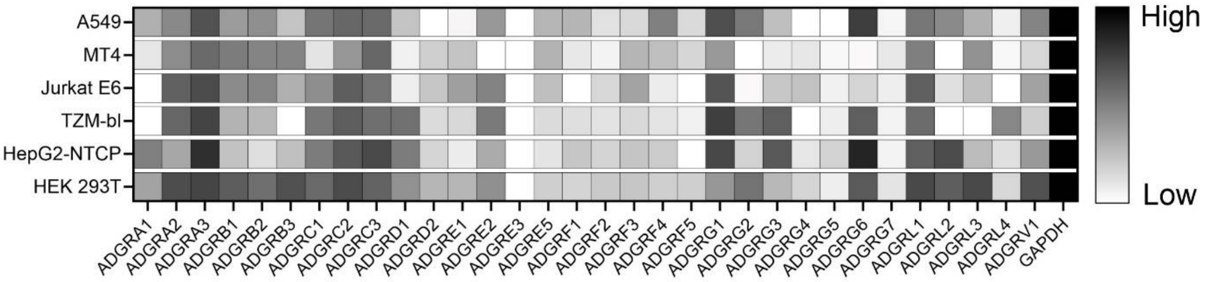

Supplementary Figure S1. Heatmap of relative mRNA expression of aGPCRs in six different cell lines. Ct values obtained by RT-qPCR analysis for individual aGPCRs were normalized to GAPDH and resulting ΔCt are presented in a heat map. Lighter shades represent low relative expression (high ΔCt values) and darker shades stand for high relative expression (low ΔCt values). A549, human pulmonary adenocarcinoma cell line; MT4, human T-lymphotropic virus I (HTLV-I) transformed human T cell line; Jurkat E6, human leukemic T-cell lymphoblast cell line; TZM-bl, HeLa cell line generated from JC.53 cells by introducing separate integrated copies of the luciferase and β-galactosidase genes under control of the human immunodeficiency virus type 1 (HIV-1) promoter; HepG2-NTCP, human liver cancer cell line, HepG2, stably transfected with the human HBV entry receptor - sodium taurocholate co-transporting polypeptide (NTCP); HEK 293T, human embryonic kidney tissue cells expressing a mutant version of the SV40 large T antigen.

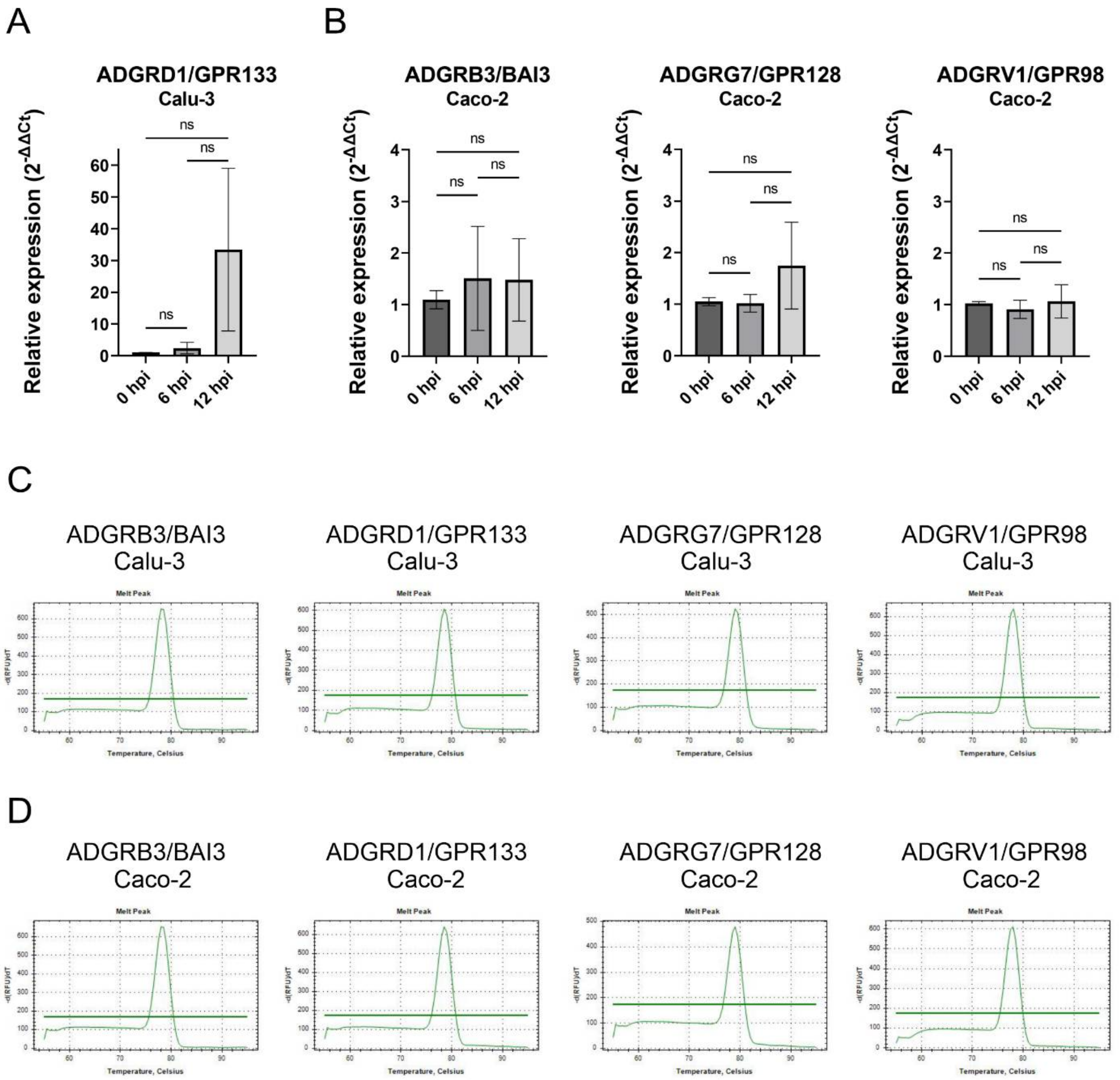

Supplementary Figure S2. Relative mRNA expression of selected aGPCRs in Calu-3 (A) and Caco-2 (B) cells that did not reach statistically significant changes of expression upon SARS-CoV-2 infection at MOI of 2. Ct values obtained for aGPCR candidates were normalized to GAPDH and resulting ΔCt values were normalized against a PBS-treated negative control. ΔΔCt values were converted to expression fold change using formula $exp = 2^{-\Delta\Delta Ct}$ and in graphs are shown as mean and standard deviation of three (Calu-3 cells) and six (Caco-2 cells) independent biological replicates performed in technical duplicates. The relative expression of aGPCRs at 6 h.p.i. and 12 h.p.i. vs 0 h.p.i. and 6 h.p.i. vs 12 h.p.i. were compared by Kruskal-Wallis (S2A) and one-way ANOVA (S2B) ($n = 3$ for Calu-3 cells and $n = 6$ for Caco-2 cells, ns - not significant) in GraphPad Prism 10.0.2. Representative melting curves of aGPCRs amplicon from RT-PCR analysis in Calu-3 (C) and Caco-2 (D) cells were analysed using Bio-Rad CFX Maestro software.

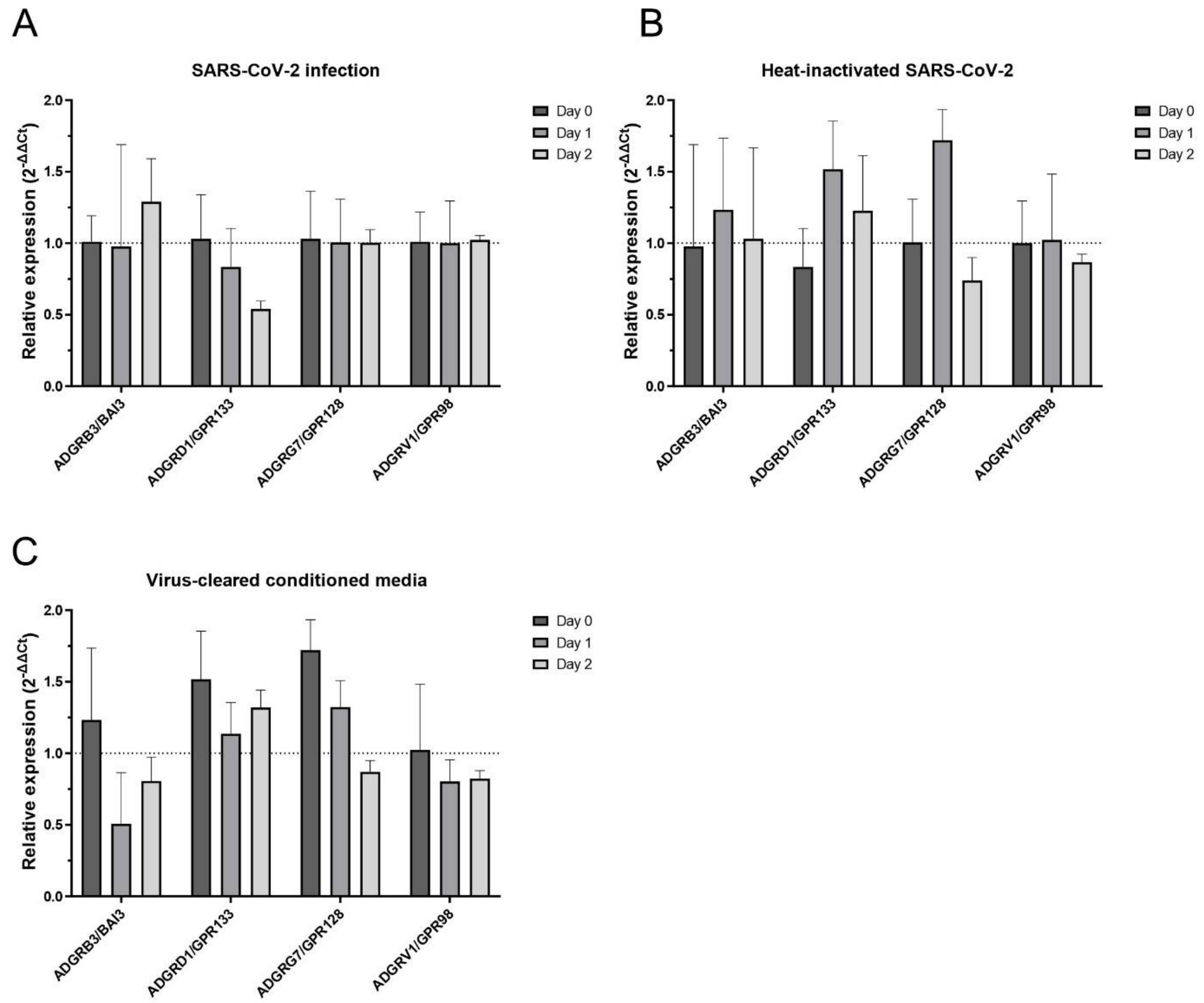

Supplementary Figure S3. Insignificant changes of relative mRNA expression of selected aGPCRs in Caco-2 cells after infection with SARS-CoV-2, exposure to heat-inactivated SARS-CoV-2 and virus-cleared conditioning media. Relative mRNA expression of selected aGPCRs in Caco-2 cells after (A) infection of SARS-CoV-2 with MOI of 0.01, (B) treatment of heat-inactivated SARS-CoV-2 and (C) virus-cleared conditioned media. Ct values obtained for aGPCR candidates were normalized against the geometric mean of GAPDH and PPIA and resulting ΔCt values were normalized against a PBS-treated negative control. ΔΔCt values were converted to expression fold change using formula exp = $2^{-\Delta\Delta Ct}$ and in graphs are shown as averages and standard deviations of three independent biological replicates.

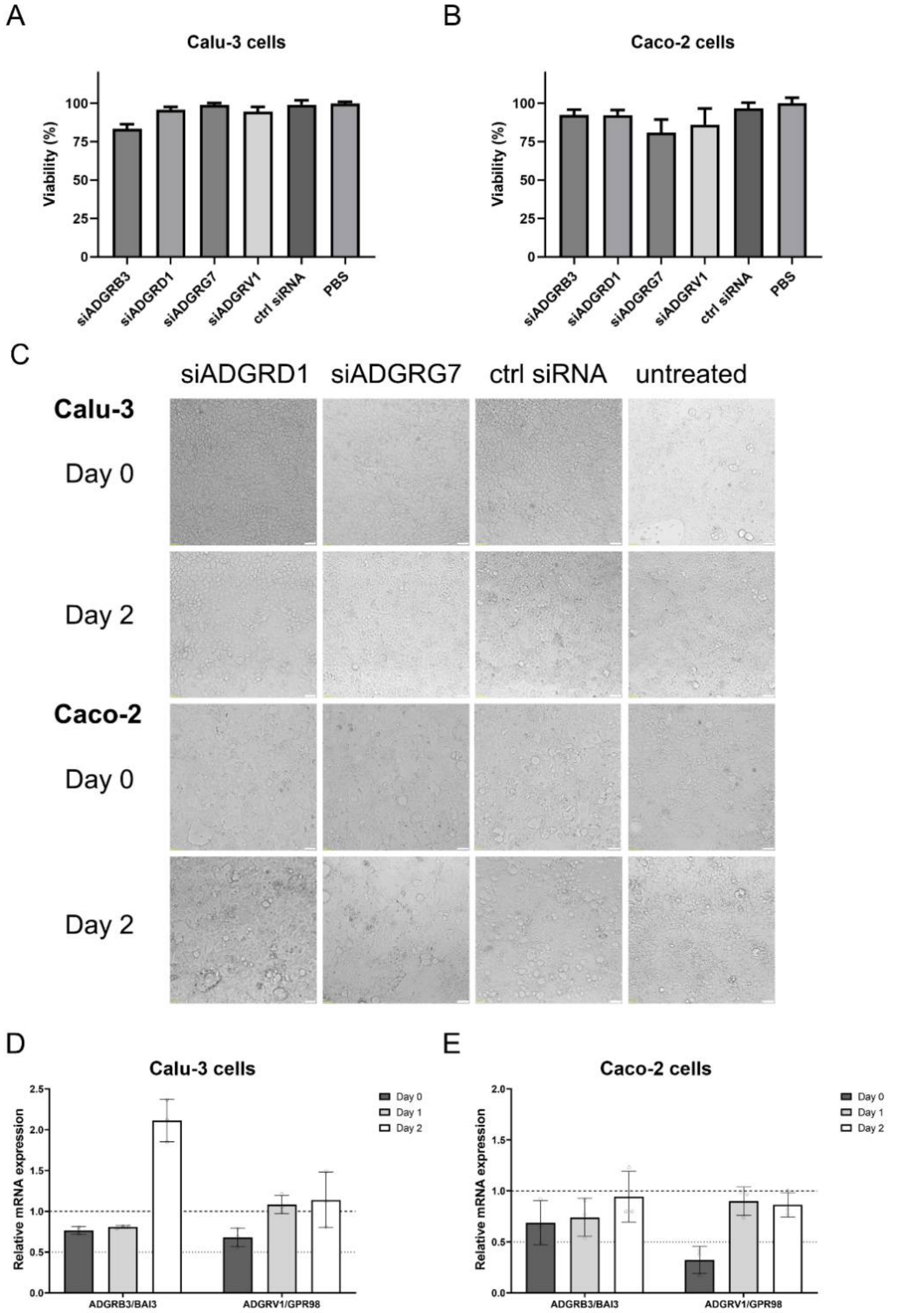

Supplementary Figure S4. Cytotoxicity of siRNAs used. Calu-3 (A) and Caco-2 (B) cells were treated with the individual siRNAs or PBS only, following the same experimental design as in Figure 3A. Cell viability was evaluated by XTT assay at day 2. (C) No changes in cell morphology were observed after aGPCR knockdown. Representative images of aGPCRs-downregulated Calu-3 and Caco-2 cells at day 0 (third day after 1. siRNA treatment) and day 2 (second day after 2. siRNA treatment). Cells were imaged with an Olympus IX81 inverted microscope (Olympus, Japan). White bars are at bottom right corners and represent 50 µm. Downregulation of endogenous ADGRB3/BAI3 and ADGRV1/GPR98 mRNA expression in Calu-3 (D) and Caco-2 (E) cells after siRNA treatment at day −3 and day 0 was estimated by RT-qPCR at day 0, day 1 and day 2. Obtained Ct values were normalized against the geometric mean of GAPDH and PPIA, and the resulting ΔCt values were normalized against an siRNA-treated negative control. ΔΔCt values were converted to expression fold-change using the formula $\Delta\Delta Ct = 2^{-\Delta\Delta Ct}$ and are presented in graphs as averages and standard deviations of three independent biological replicates.

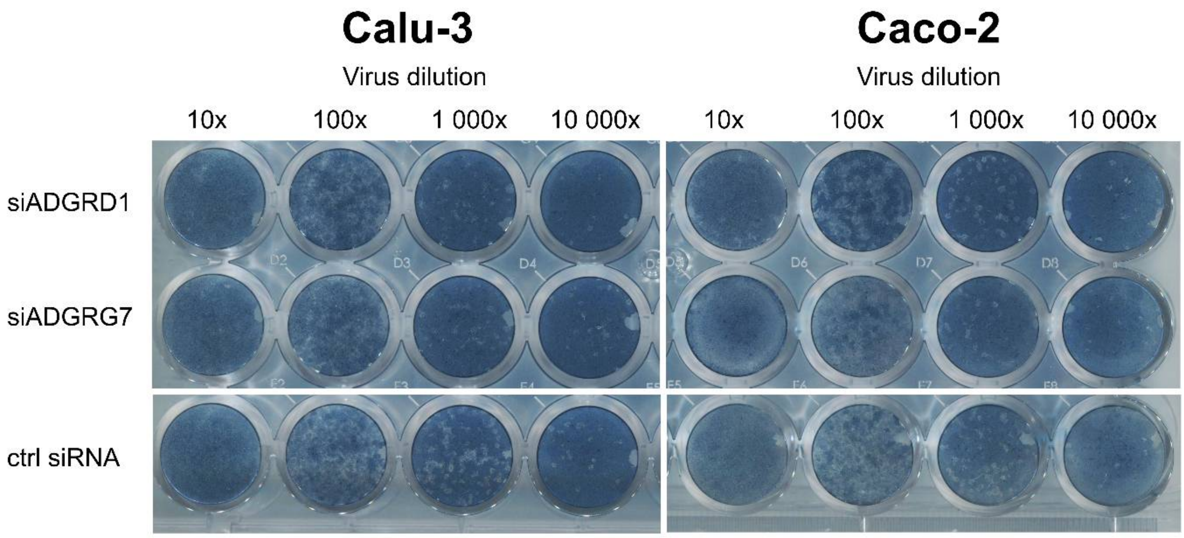

Supplementary Figure S5. Representative figures of plaque assays at 48 h.p.i.. The cultivation media from aGPCRs-downregulated SARS-CoV-2-infected Calu-3 and Caco-2 cells were analysed in a plaque assay in Vero E6 cells. Supernatants were titrated in 10-fold dilution steps from 10× to 10,000×.

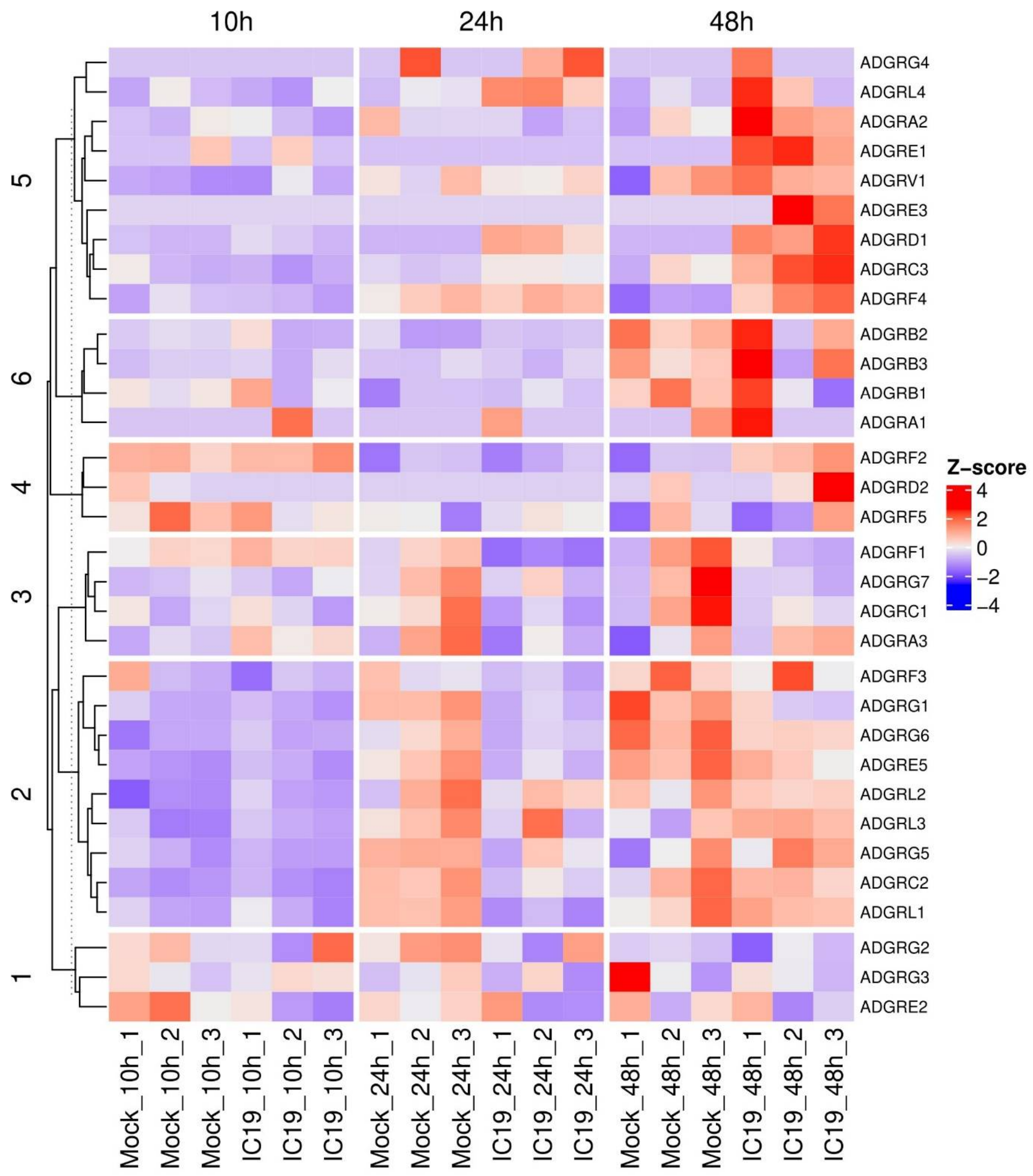

Supplementary Figure S6. Comparison of adhesion GPCR expression in non-infected Calu-3 cells and cells after infection with SARS-CoV-2 (IC19 strain) from dataset GSE213759 [1]. The heatmap represents TPM normalized expression levels observed 10, 24 and 48 h after infection performed in triplicate. Adhesion GPCRs are organized into 6 clusters based on their similarity in the expression profile.

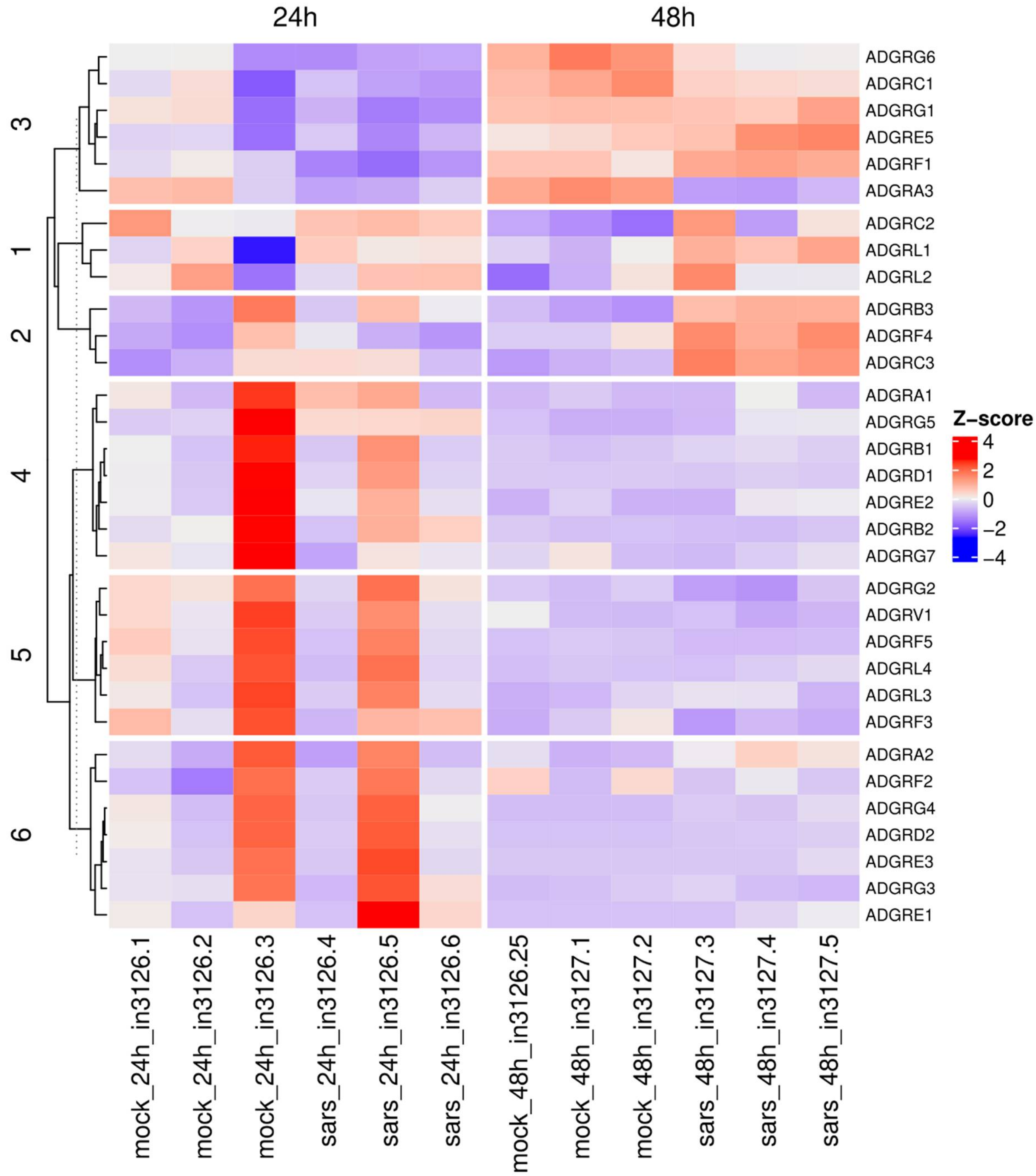

Supplementary Figure S7. Comparison of adhesion GPCR expression in non-infected Calu-3 cells and cells after infection with SARS-CoV-2 (WA1 strain) from dataset GSE252056 [2]. The heatmap represents TPM normalized expression levels observed 24 and 48 h after infection performed in triplicate. Adhesion GPCRs are organized into 6 clusters based on their similarity in the expression profile.

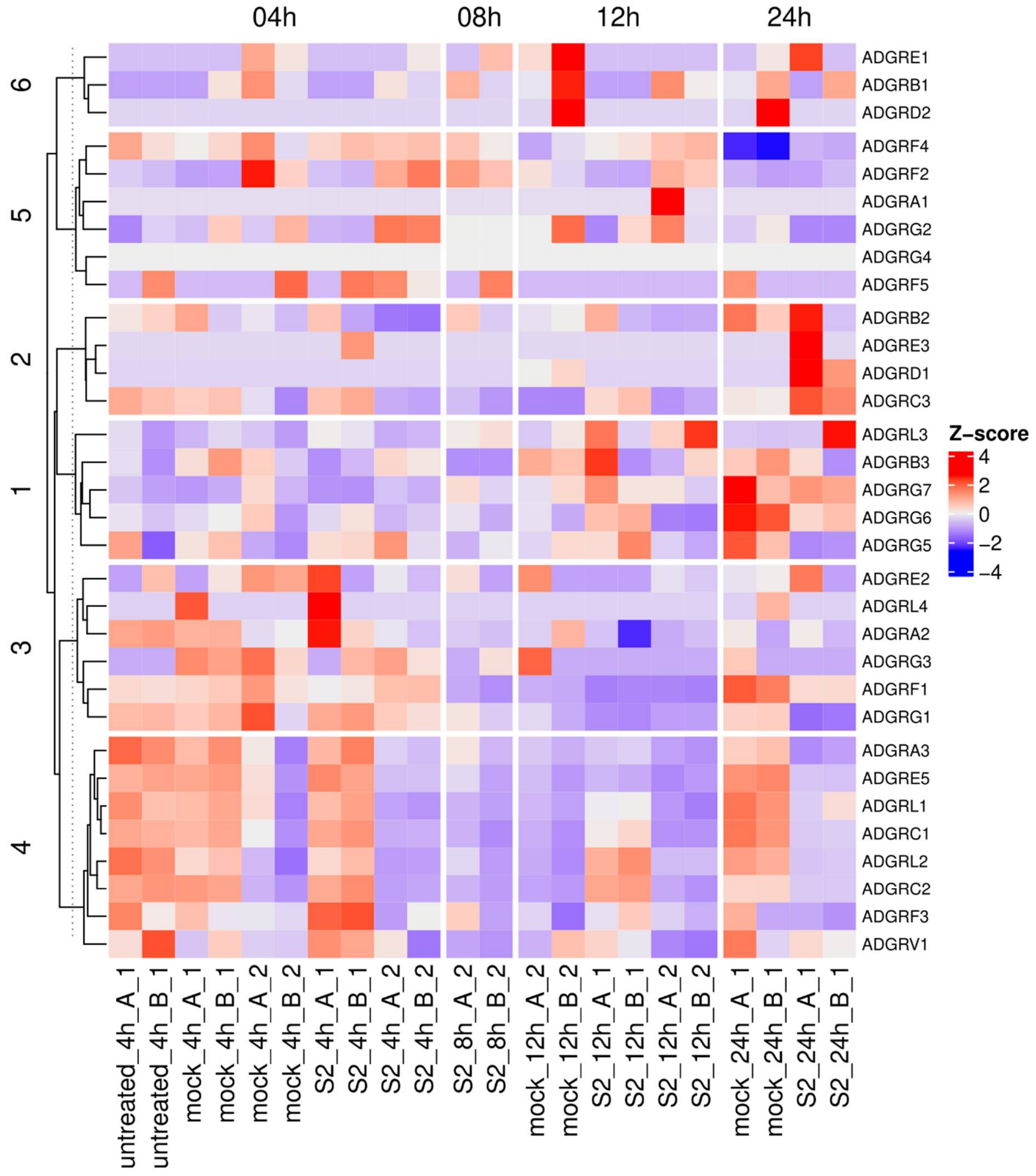

Supplementary Figure S8. Comparison of adhesion GPCR expression in non-infected Calu-3 cells and cells after infection with SARS-CoV-2 (strain hCoV-19/Germany/BY-ChVir-929/2020) from dataset GSE148729 [3]. The heatmap represents TPM normalized expression levels observed 4, 8, 12 and 24 h after infection in two biological replicates (4, 12 and 24 h labelled as 1, 4, 8 and 12 h labelled as 2) performed in duplicates (labelled as A and B). Adhesion GPCRs are organized into 6 clusters based on the similarity in expression profile.

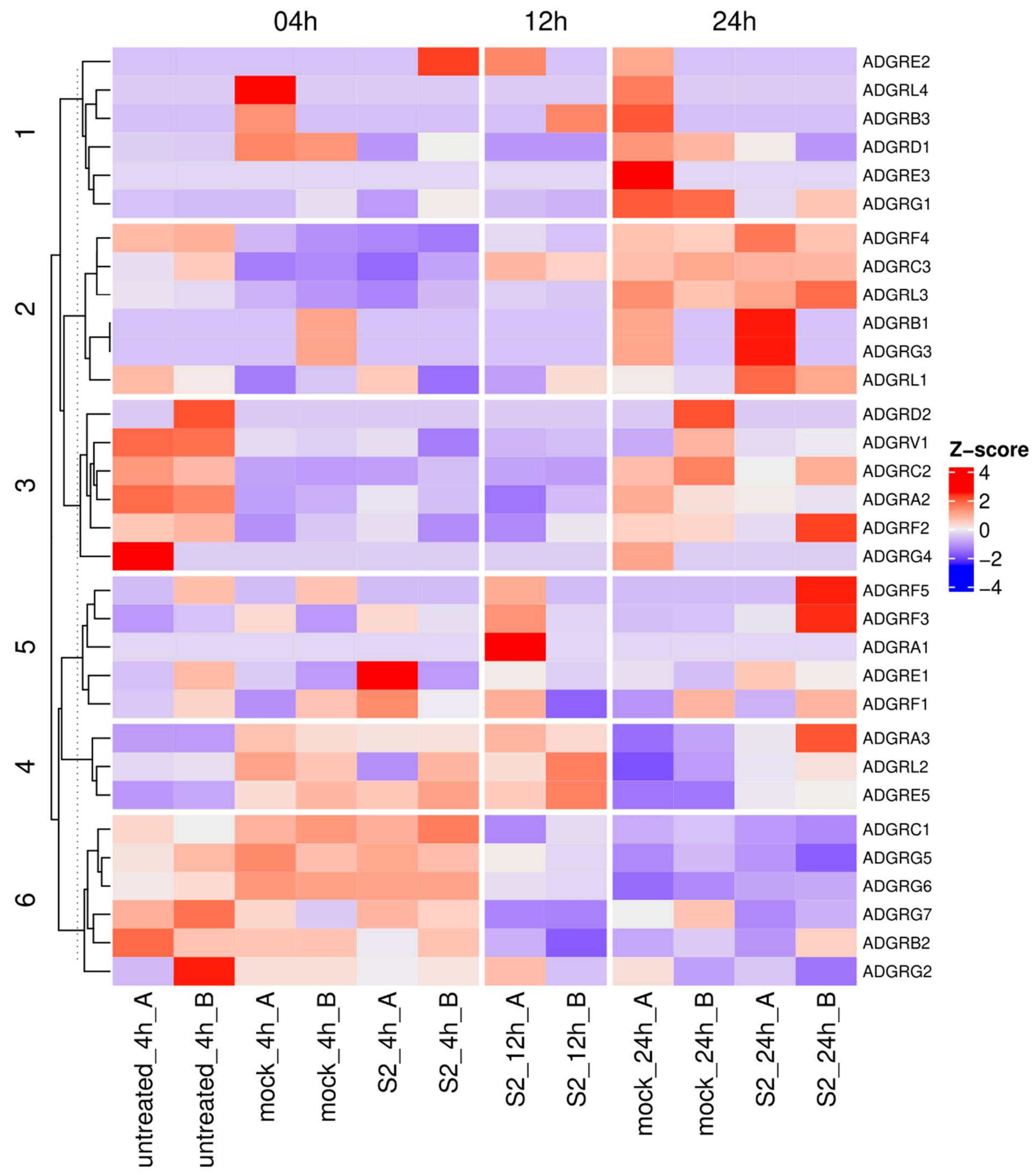

Supplementary Figure S9. Comparison of adhesion GPCR expression in non-infected Caco-2 cells and cells after infection with SARS-CoV-2 (strain hCoV-19/Germany/BY-ChVir-929/2020) from dataset GSE148729 [3]. The heatmap represents TPM normalized expression levels observed 4, 12 and 24 h after infection performed in duplicates (labelled as A and B). Adhesion GPCRs are organized into 6 clusters based on their similarity in the expression profile.

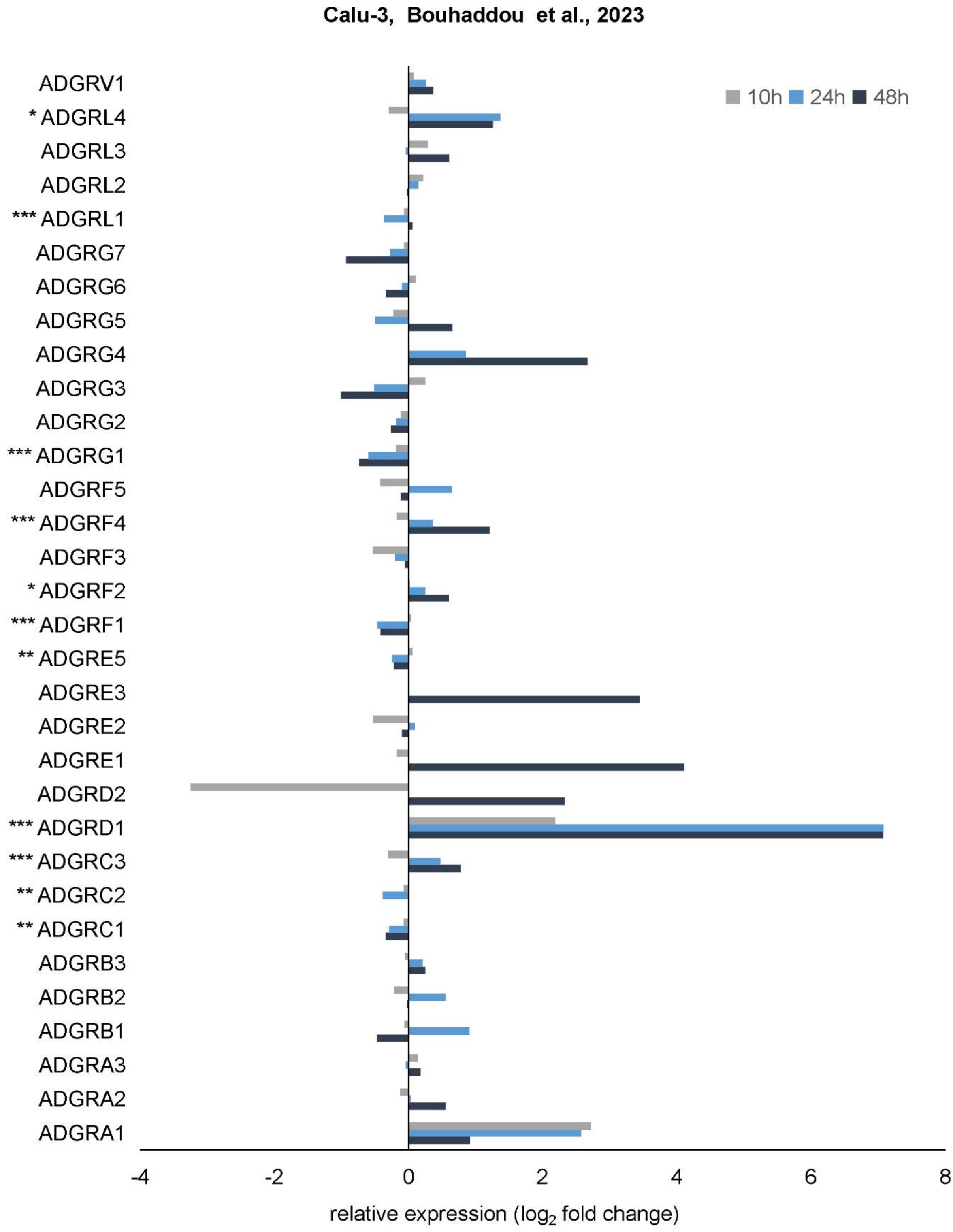

Supplementary Figure S10. Relative gene expression of adhesion GPCRs in Calu-3 cells 10, 24 and 48 h after infection with SARS-CoV-2 (IC19 strain) from dataset GSE213759 [1]. Horizontal bars show relative expression in infected cells vs controls calculated using DESeq2 statistic package. Asterisks represent statistically significant results with a false discovery rate of 0.05 (*), 0.01 (**) and 0.001 (***) for at least one time point.

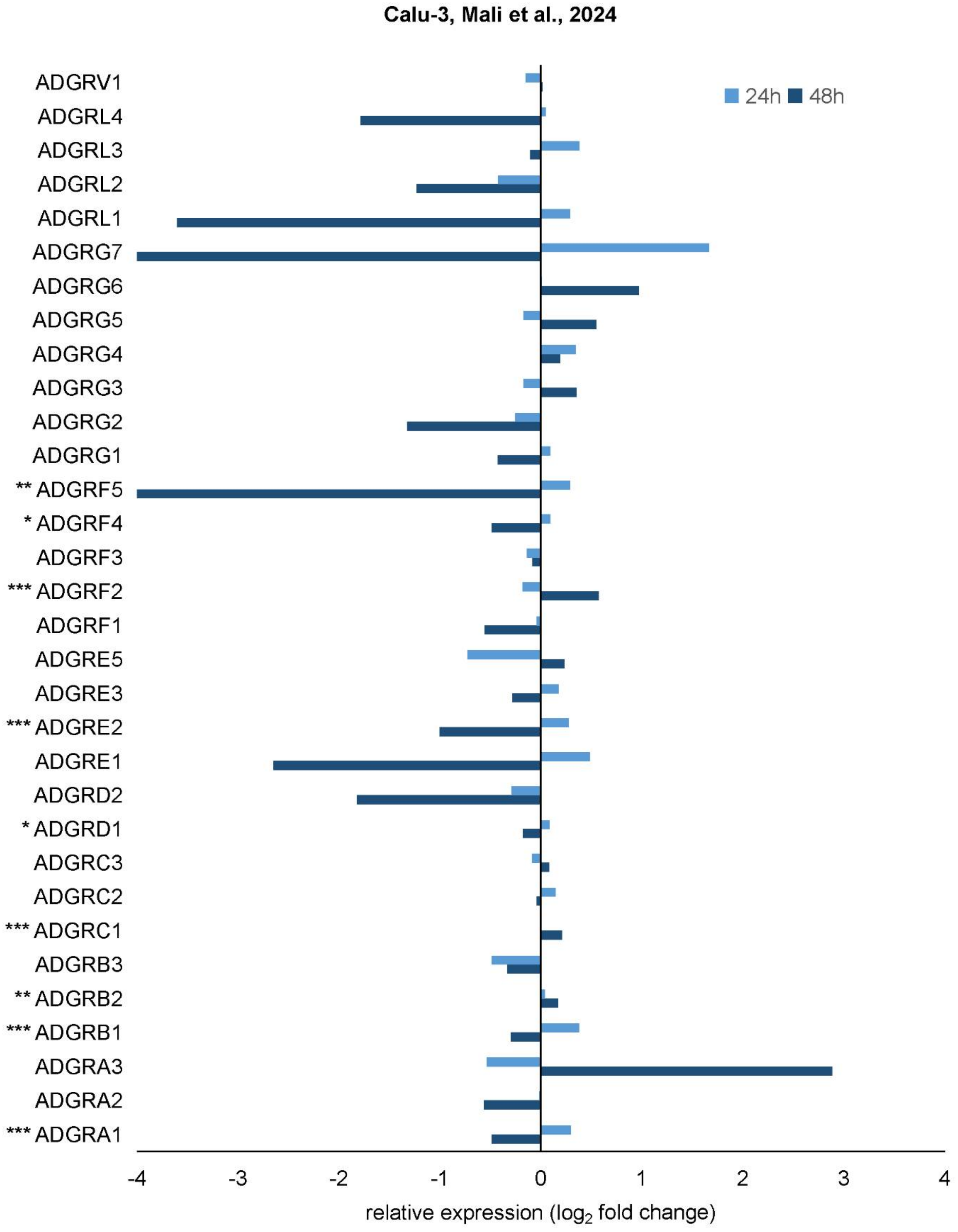

Supplementary Figure S11. Relative gene expression of adhesion GPCRs in Calu-3 cells 24 and 48 h after infection with SARS-CoV-2 (WA1 strain) from dataset GSE252056 [2]. Horizontal bars show relative expression in infected cells vs controls calculated using DESeq2 statistic package. Asterisks represent statistically significant results with a false discovery rate of 0.05 (*), 0.01 (**) and 0.001 (***) for at least one time point.

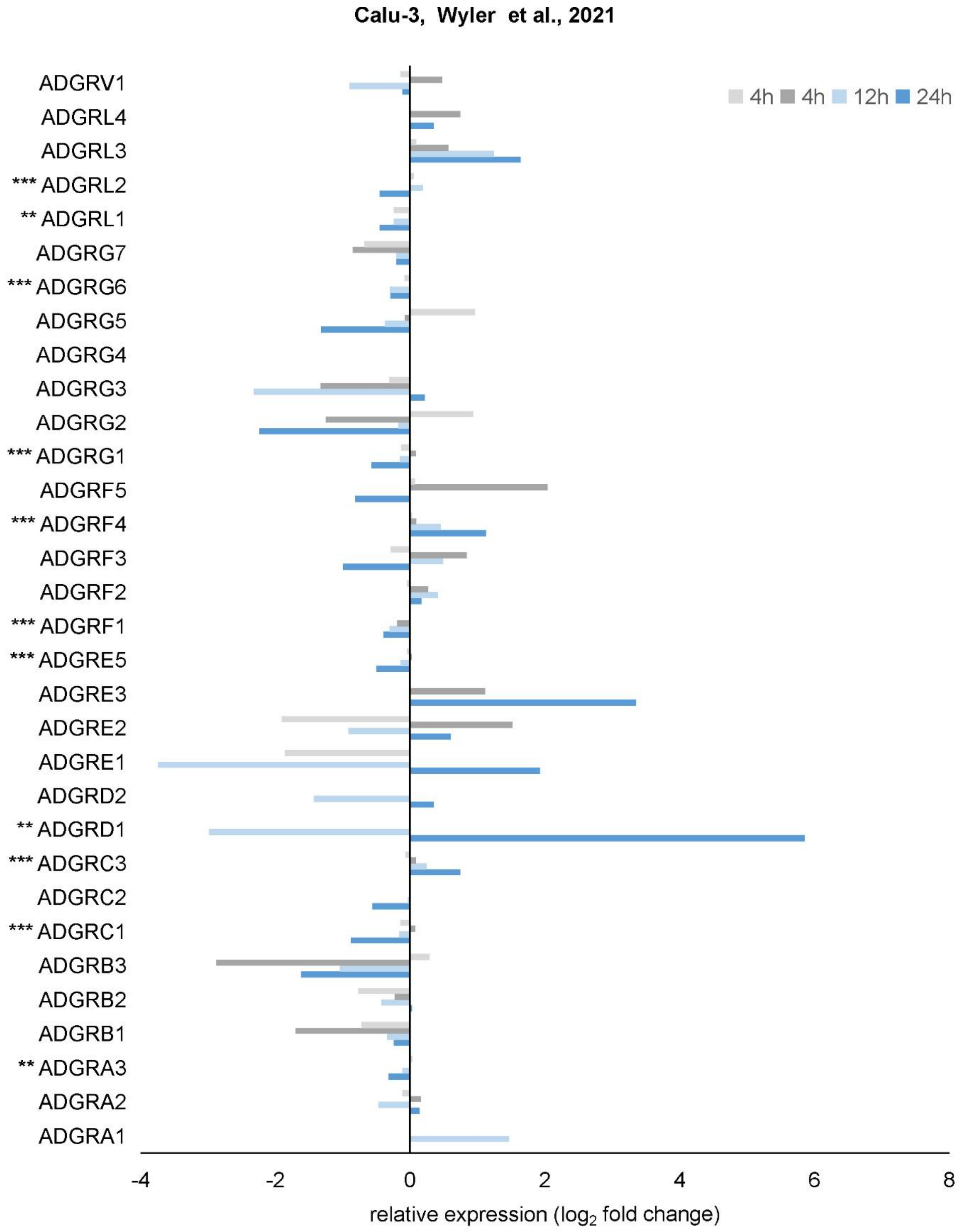

Supplementary Figure S12. Relative gene expression of adhesion GPCRs in Calu-3 cells 4, 12 and 24 h after infection with SARS-CoV-2 (strain hCoV-19/Germany/BY-ChVir-929/2020) from dataset GSE148729 [3]. It includes timepoints 4, 12 and 24 h after infection from 1st experiment and timepoint 4 h (light grey) after infection from 2nd experiment. Relative gene expression for 8 and 12 h after infection from 2nd experiment could not be determined because of missing data from mock conditions. Horizontal bars show relative expression in infected cells vs controls calculated using DESeq2 statistic package. Asterisks represent statistically significant results with a false discovery rate of 0.01 (**) and 0.001 (***) for at least one time point.

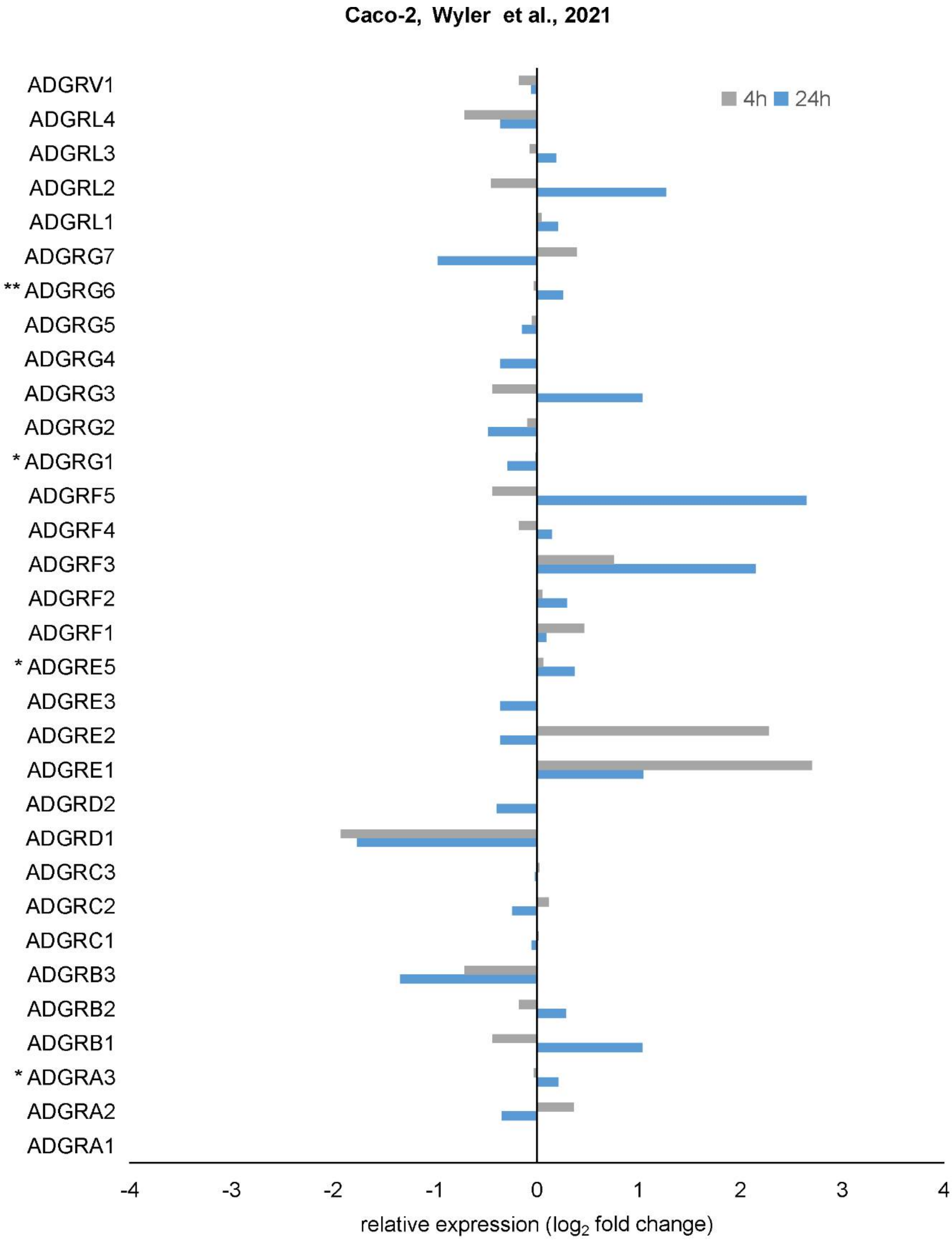

Supplementary Figure S13. Relative gene expression of adhesion GPCRs in Caco-2 cells 4 and 24 h after infection with SARS-CoV-2 (strain hCoV-19/Germany/BY-ChVir-929/2020) from dataset GSE148729 [3]. Horizontal bars show relative expression in infected cells vs controls calculated using DESeq2 statistic package. Asterisks represent statistically significant results with a false discovery rate of 0.05 (*) and 0.01 (**) for at least one time point.

References

1. Bouhaddou, M.; Reuschl, A.K.; Polacco, B.J.; Thorne, L.G.; Ummadi, M.R.; Ye, C.; Rosales, R.; Pelin, A.; Batra, J.; Jang, G.M.; et al. SARS-CoV-2 variants evolve convergent strategies to remodel the host response. *Cell* **2023**, *186*, 4597–4614 e4526. https://doi.org/10.1016/j.cell.2023.08.026.
2. Mali, S.S.; Silva, R.; Gong, Z.; Cronce, M.; Vo, U.; Vuong, C.; Moayedi, Y.; Cox, J.S.; Bautista, D.M. SARS-CoV-2 papain-like protease activates nociceptors to drive sneeze and pain. *bioRxiv* **2024**, 10.1101/2024.01.10.575114. https://doi.org/10.1101/2024.01.10.575114.
3. Wyler, E.; Mosbauer, K.; Franke, V.; Diag, A.; Gottula, L.T.; Arsie, R.; Klironomos, F.; Koppstein, D.; Honzke, K.; Ayoub, S.; et al. Transcriptomic profiling of SARS-CoV-2 infected human cell lines identifies HSP90 as target for COVID-19 therapy. *iScience* **2021**, *24*, 102151. https://doi.org/10.1016/j.isci.2021.102151.
